# Targeting BCL11B in CAR-engineered lymphoid progenitors drives NK-like cell development with prolonged anti-leukemic activity

**DOI:** 10.1016/j.ymthe.2025.02.024

**Published:** 2025-02-15

**Authors:** Franziska Baatz, Arnab Ghosh, Jessica Herbst, Saskia Polten, Johann Meyer, Manuel Rhiel, Tobias Maetzig, Robert Geffers, Michael Rothe, Antonella Lucia Bastone, Philipp John-Neek, Jörg Frühauf, Britta Eiz-Vesper, Agnes Bonifacius, Christine S. Falk, Constantin v. Kaisenberg, Toni Cathomen, Axel Schambach, Marcel R.M. van den Brink, Michael Hust, Martin G. Sauer

**Affiliations:** 1 Department of Pediatric Hematology, Department of Oncology and Blood Stem Cell Transplantation, Hannover Medical School, Hannover, Germany; 2 Adult BMT Service, Department of Medicine, Memorial Sloan Kettering Cancer Center, New York, NY, USA; 3 Department of Medical Biotechnology, Technische Universität Braunschweig, Braunschweig, Germany; 4 Institute of Experimental Hematology, Hannover Medical School, Hannover, Germany; 5 Institute for Transfusion Medicine and Gene Therapy, Medical Center-University of Freiburg, Freiburg, Germany; 6 Genome Analytics, Helmholtz Centre for Infection Research, Braunschweig, Germany; 7 REBIRTH - Research Center for Translational Regenerative Medicine, Hannover Medical School, Hannover, Germany; 8 Clinic for Radiation Therapy and special Oncology, Hannover Medical School, Hannover, Germany; 9 Institute for Transfusion Medicine, Hannover Medical School, Hannover, Germany; 10 Institute of Transplant Immunology, Hannover Medical School, Hannover, Germany; 11 Department of Obstetrics, Clinic of Gynecology and Reproductive Medicine, and Obstetrics, Hannover Medical School, Hannover, Germany; 12 Center for Chronic Immunodeficiency (CCI), Medical Center-University of Freiburg, Freiburg, Germany; 13 Faculty of Medicine, University of Freiburg, Freiburg, Germany; 14 Division of Hematology/Oncology, Boston Children’s Hospital, Harvard Medical School, Boston, MA, USA; 15 City of Hope National Medical Center, Duarte, CA, USA

**Keywords:** hematopoietic stem cell transplantation, lymphoid progenitors, BCL11B, gene editing, NK cell differentiation, adoptive immunotherapy, acute myeloid leukemia

## Abstract

Chimeric antigen receptor (CAR)-induced suppression of the transcription factor B cell CLL/lymphoma 11B (BCL11B) propagates CAR-induced killer (CARiK) cell development from lymphoid progenitors. Here, we show that CRISPR-Cas9-mediated *Bcl11b* knockout in human and murine early lymphoid progenitors distinctively modulates this process either alone or in combination with a CAR. Upon adoptive transfer into hematopoietic stem cell recipients, *Bcl11b*-edited progenitors mediated innate-like antigen-independent anti-leukemic immune responses. With CAR expression allowing for additional antigen-specific responses, the progeny of double-edited lymphoid progenitors acquired prolonged anti-leukemic activity *in vivo*. These findings give important insights into how *Bcl11b* targeting can be used to tailor anti-leukemia functionality of CAR-engineered lymphoid progenitor cells.

## Graphical abstract

**Figure:**
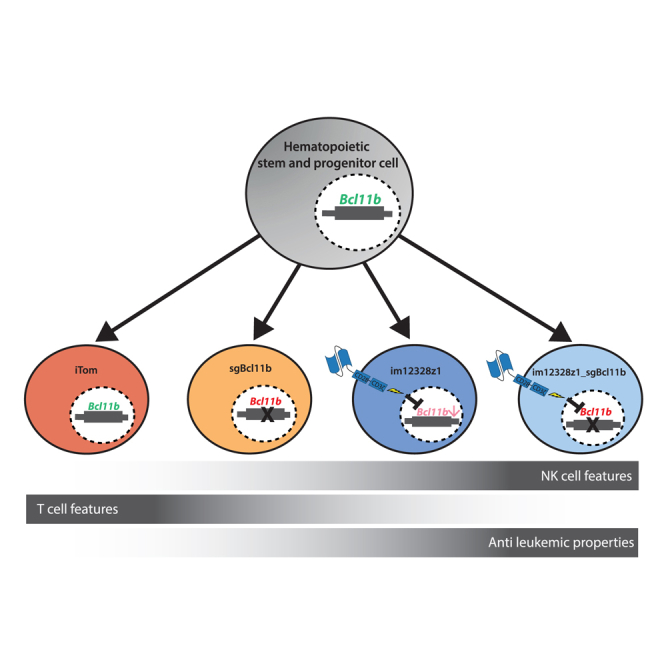

## Teaser

Sauer and colleagues demonstrate that *Bcl11b*-edited lymphoid progenitors mediate NK-like anti-leukemic activity. Adding a chimeric antigen receptor (CAR) gives rise to a distinct CAR-induced killer cell population with prolonged anti-leukemic efficacy. These findings give important insights into how *Bcl11b* targeting can be used to tailor anti-leukemia functionality of CAR-engineered lymphoid progenitors.

## Introduction

Relapse after allogeneic hematopoietic stem cell transplantation (HSCT) is a major cause of mortality in acute myeloid leukemia (AML). In an effort to decrease the likelihood of relapse, we and others have explored a strategy to enrich a previously T cell-depleted hematopoietic stem cell (HSC) graft with *in vitro* chimeric antigen receptor (CAR)-engineered lymphoid progenitor cells.^1^^,^^2^^,^^3^^,^^4^

The transcription factor B cell CLL/lymphoma 11B (BCL11B) has been described to function as a transcriptional activator and repressor.^5^^,^^6^ It acts as a pivotal regulator during thymocyte maturation and later regulates the function and differentiation of mature T cells.^7^^,^^8^^,^^9^^,^^10^^,^^11^^,^^12^^,^^13^ The lack of BCL11B in Th17 cells derepresses the Th2 program.^14^ In contrast, its absence in Th2 cells reduces their ability to differentiate in response to asthma and helminth infection.^15^ BCL11B in regulatory T cells prevents devastating autoimmunity.^16^^,^^17^ In ILC2 cells, it is fundamental for development and maintenance of identity^18^^,^^19^^,^^20^ and BCL11B depletion in naive CD8^+^ T cells results in a failure to raise antigen-specific responses and undergo clonal expansion.^21^^,^^22^ BCL11B was recently shown to be required for the effector functions of intestinal resident memory CD8^+^ T cells.^23^ Of note, BCL11B promotes differentiation of canonical and adaptive natural killer (NK) cells.^24^ Although the majority of these studies were conducted in mice and similar mechanisms were reported in human lymphoid cell lineage development,^24^^,^^25^^,^^26^ recent work has uncovered a distinctive role of BCL11B in human NK cell differentiation.

Using a cell culture system that at least partially mimics the NOTCH-driven T cell developmental process,^27^ we had previously shown that CAR-mediated signaling during early phases of progenitor development downregulated BCL11B, leading to a suppression of T cell-associated gene expression and acquisition of NK cell-like properties. After co-transplantation with T cell-depleted HSCs into transplant recipients, anti-CD19 CAR-expressing lymphoid progenitors differentiated into CAR-induced killer (CARiK) cells that mediated potent antigen-directed anti-leukemic activity even across major histocompatibility complex (MHC) barriers.^4^ However, this developmental shift required either significant tonic signaling activity by the CAR or continuous antigen-specific stimulation during differentiation.

Here, we sought to assess whether CARiK cells could be generated by *Bcl11b* disruption in combination with more clinically relevant CARs against AML that have reduced tonic signaling activity. Starting with a CAR against human CD123 (h12328bbz) in umbilical cord blood (UCB)-derived lymphoid progenitors *in vitro*, we assessed the *in vivo* significance of an additional *Bcl11b* disruption in an immunologically coherent murine model. In accordance with earlier reports, we demonstrate that *Bcl11b* knockout during early lymphoid development skews differentiation to a population with NK-like properties.^8^^,^^11^^,^^28^^,^^29^ In combination with CAR expression, we obtained a CARiK cell population with more pronounced NK cell-like features that provided longer activity *in vivo*, thereby protecting from a modeled late relapse. These findings are clinically relevant in that *Bcl11b* editing could be used to tailor CARiK cells for more durable anti-tumor efficacy.

## Results

### Isolated *BCL11B* knockdown in cord blood-derived CD34^+^ HSPCs inhibit T cell differentiation *in vitro* less profoundly than early hCD123-CAR expression

**Figure 1:**
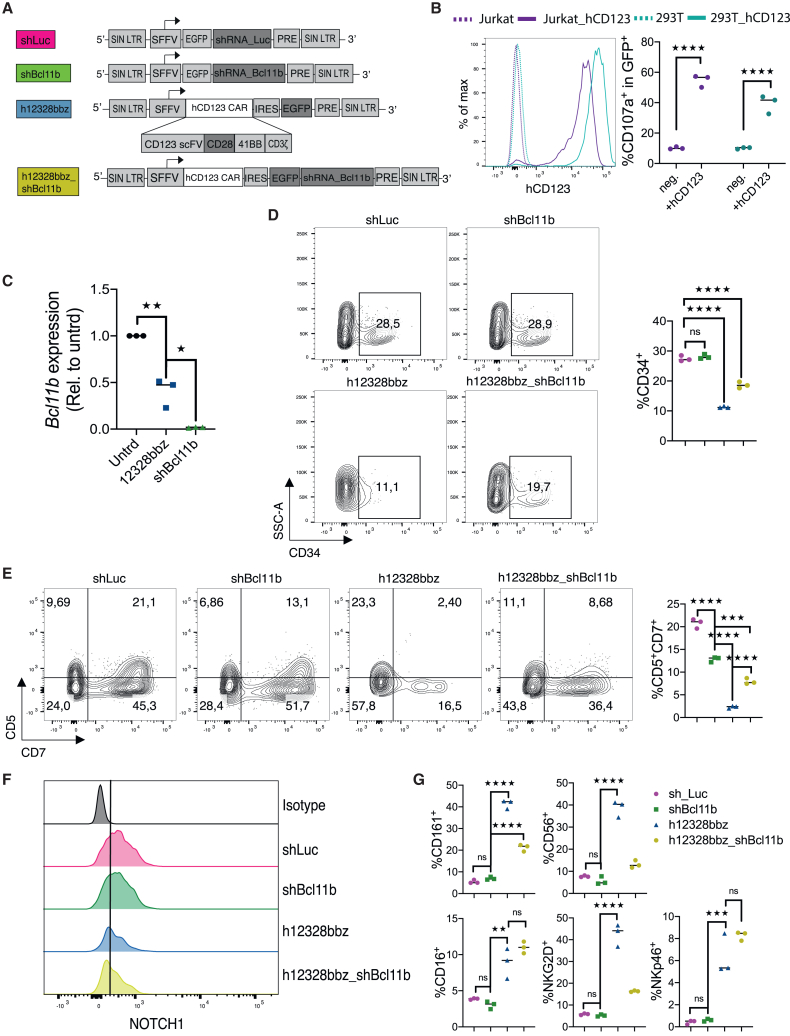
Isolated *BCL11B* knockdown in cord-blood-derived CD34^+^ HSPCs inhibits T cell differentiation *in vitro* less profoundly than early hCD123-CAR expression (A) Representation of the lentiviral vectors: shLuc (luciferase-targeted shRNA used as control), shBcl11b (*Bcl11b*-targeted shRNA), h12328bbz1 (human CD123 CAR), and h12328bbz1_shBcl11b (human CD123 CAR + *Bcl11b*-targeted shRNA). All constructs are equipped with a GFP reporter gene. (B) Responses of h12328bbz-transduced PBMCs upon stimulation with either naive or hCD123-transduced Jurkat or 293T cells. Stimulation was quantified by measuring CD107a degranulation. Representative data from one of three independent experiments are shown. (C–G) Human CD34^+^ UCB-derived HSPCs were engineered with the respective constructs and consecutively differentiated on OP9-DL1 stromal cells. Expression and flow cytometry analysis were performed within the GFP^+^ gate on day 14 of co-culture. Representative results from one of two independent experiments are shown. (C) qPCR analysis of *Bcl11b* expression in transduced lymphoid progenitors. (D and E) Representative flow cytometry analysis of engineered human hematopoietic progenitors showing different stages of T cell development as determined by CD5, CD7, and CD34 expression. (F) Histograms represent NOTCH1 expression on respectively engineered lymphoid progenitors on day 14 of culture. (G) Lymphoid progenitors derived from modified HSPCs were comparatively analyzed for NKG2D, NKp46, CD16, and CD161 expression on day 14 of culture. Data from one experiment are shown. Statistics were performed using Student’s t test (two tailed) and one-way ANOVA with Tukey’s *post hoc* test. Each data point represents an individual sample. ∗*p* < 0.05; ∗∗*p* < 0.01; ∗∗∗*p* < 0.001; ∗∗∗∗*p* < 0.0001; ns, not significant.

Using a human CAR against human CD123 (h12328bbz), we sought to assess whether a direct *BCL11B* knockdown in lymphoid progenitors could comparatively recapitulate a differentiation shift from T to NK cell fate as reported earlier,^11^^,^^30^ and whether an additional *BCL11B* knockdown in CAR-expressing progenitors would further impact NK cell-like development (Figure 1A). For *BCL11B*-knockdown experiments, we screened four different *BCL11B*-targeted short hairpin RNAs (shRNAs) and chose the most efficient shBcl11b_3446 for further experiments (Figures S1A and S1B). HSPCs transduced with a non-targeting shRNA against luciferase served as non-specific controls. First, we assessed whether h12328bbz was responsive upon specific antigen stimulation. Human peripheral blood mononuclear cells (PBMCs) were transduced with h12328bbz and stimulated with either 293T or Jurkat target cells that were engineered to overexpress the human CD123 protein. Wild-type (WT) 293T and Jurkat cells served as negative controls. Antigen stimulation resulted in specific responses of h12328bbz-transduced PBMCs as measured by degranulation and CD107a expression (Figure 1B), proving functionality and antigen specificity. Next, human UCB-derived CD34^+^ HSPCs were transduced with a *BCL11B*-targeted shRNA (shBcl11b), h12328bbz, an all-in-one vector for CAR expression and *BCL11B* knockdown (h12328bbz_shBcl11b), or with the luciferase control (shLuc) (Figure 1A) and consecutively co-cultured on OP9-DL1 monolayers, which had been supplemented with human interleukin (IL)-7 (hIL-7), hSCF, hTPO, and hFLT3L to induce lymphoid progenitor cell differentiation. Bcl11b expression in lymphoid progenitors was significantly suppressed after CAR transduction and nearly completely eliminated after shBcl11b transduction as shown by reverse-transcription qPCR (Figure 1C). h12328bbz expression resulted in declining frequencies of CD34^+^ and CD7^+^CD5^+^ cells (Figures 1D and 1E). This was associated with decreased NOTCH1 expression (Figure 1F) and increased frequencies of NKp46^+^, NKG2D^+^, CD16^+^, CD56^+^, or CD161^+^ cells, suggesting a block of T cell in favor of NK cell differentiation (Figure 1G). Direct *BCL11B* knockdown only recapitulated this switch incompletely. It blocked T development at this stage of *in vitro* culture less rigorously (Figures 1D and 1E), mediated little if any suppression of NOTCH1 (Figure 1F), and initiated no significant upregulation of NK markers (Figure 1G). T cell receptor (TCR)-β expression was not detectable at this early stage of development (day 14), whereas low γδTCR expression occurred; however, this was comparable between groups (Figure S1C). The double-engineered progenitors demonstrated a more intermediate phenotype in regard to early T cell marker suppression (Figures 1D and 1E). Frequencies of CD16^+^ and NKp46^+^ cells were elevated (Figure 1G) and NOTCH1 expression downregulated (Figure 1F). This was comparable to CAR-only edited progenitors. The frequency of CD161-expressing cells, however, was higher in CAR-only engineered UCB-derived progenitors (Figure 1F). To better understand the impact of the engineering strategies on further *in vivo* differentiation and function, we continued the studies in an immunologically coherent immunocompetent mouse model.

### Generation of a CAR against murine CD123 (im12328z1)

**Figure 2:**
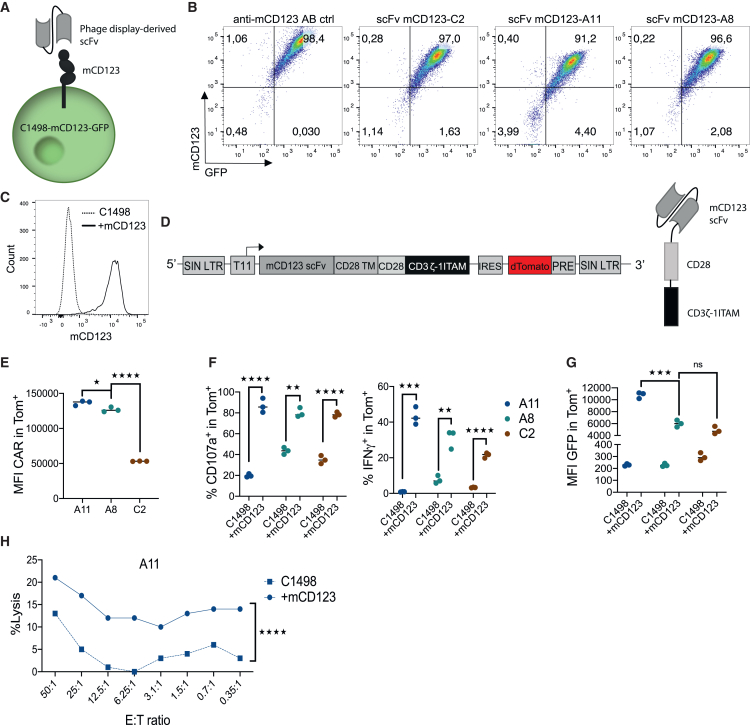
Generation of a CAR against murine CD123 (mCD123 CAR, im12328z1) (A) Antigen binders against murine CD123 were identified using HAL9/10 antibody phage display libraries.^31^ (B) His-tagged scFv binding of cell-bound mCD123 was validated by flow cytometry. Representative flow cytometry panels depict the three most promising binders, namely C2, A11, and A8. They demonstrated a binding pattern similar to the positive control that was generated with a commercially available mCD123 antibody (APC; catalog 4330936; clone 5B11). Experiments were performed once. (C) The murine AML cell line C1498 was transduced to overexpress murine CD123 and was used for im12328z1 stimulation. A representative histogram from one of three independent experiments is shown. (D) The selected three scFv binders (SH1836b-A11, -A8, and -C2) were cloned into a second-generation CAR backbone. The lentiviral CD123 CAR construct im12328z1 (inducible murine CD123 CAR, CD28 co-stimulation, one functional ITAM containing CD3ζ domain) was linked to an IRES dTomato cassette for easy detection. SIN LTR, self-inactivating long terminal repeats; T11, Dox-inducible promoter; scFv, single-chain variable fragment; TM, transmembrane domain; IRES, internal ribosome entry site; PRE, woodchuck hepatitis virus posttranscriptional regulatory element. (E–H) Comparative functionality testing of the three im12328z1 CAR constructs made with the selected three binders. Representative data from one of three independent experiments are shown. (E) im12328z1 expression (carrying the SH1836b-A11, -A8, or -C2 scFv) was assessed by flow cytometry on transduced NFAT cells using protein L staining. (F) Responses of im12328z1-transduced primary murine T cells to stimulation with either C1498-mCD123 or C1498 control cells were quantified via CD107a degranulation (left) and IFN-γ production (right). (G) Intracellular signal strength (GFP expression) of im12328z1-transduced NFAT cells upon stimulation with either C1498-mCD123 or C1498 control cells was assessed by flow cytometry. (H) Specific cytotoxic activity of im12328z1 (A11)-engineered primary murine T cells against either C1498-mCD123 or C1498 control cells using the JAM assay. MFI, mean fluorescence intensity. Statistics were performed using Student’s t test (two tailed) and one-way ANOVA with Tukey’s *post hoc* test. Each data point represents an individual sample. ∗*p* < 0.05; ∗∗*p* < 0.01; ∗∗∗*p* < 0.001; ∗∗∗∗*p* < 0.0001; ns, not significant.

In order to use an immunologically coherent murine model, we first had to generate a murine CAR against murine CD123. Therefore, we first identified suitable antibody-derived single-chain variable fragments (scFvs) against the murine CD123 antigen (mCD123) by phage display technology from the human naive antibody gene libraries HAL9/10.^31^ Binders of mCD123 were subsequently screened using the murine CD123^+^ myeloid leukemia cell line C1498-mCD123-GFP as target (Figures 2A–2C). Three binders (SH1836b-C2, -A11, -A8) were selected and cloned into a murine second-generation CAR backbone containing a CD28 co-stimulatory domain, a CD3ζ stimulatory domain, and one functional ITAM, termed im12328z1 (Figure 2D). In order to maintain comparability to our earlier work^4^ and to avoid variability caused by a different promoter, CAR expression was set again under the control of a tetracycline-inducible (Tet-on) T11 promoter and linked to an IRES-controlled dTomato (iTom) reporter cassette (Figures 2D and S2A). For inducible transgene expression, murine spleen-derived T cells with an rtTA-M2 transactivator knockin were used for all following experiments. Out of the generated three CARs, im12328z1-A11 showed the highest expression level (Figure 2E). In addition, stimulation of im12328z1-A11-transduced murine primary T cells with mCD123-expressing target cells *in vitro* induced a higher degree of specific activation when compared to SH1836b-C2 and -A8. (Figure 2F). Finally, im12328z1-A11 showed the strongest antigen-induced signaling activity and low tonic background activation in the NFAT-inducible GFP reporter system (Figures 2G and S2A–S2E). Since the im12328z1-A11 construct mediated clear antigen-dependent cytotoxic activity against C1498-mCD123 cells as well (Figure 2H), it was therefore selected for further experiments.

### im12328z1-engineered HSPCs differentiate into CARiK cells *in vitro* and provide strong anti-leukemia effects in HCT recipients

**Figure 3:**
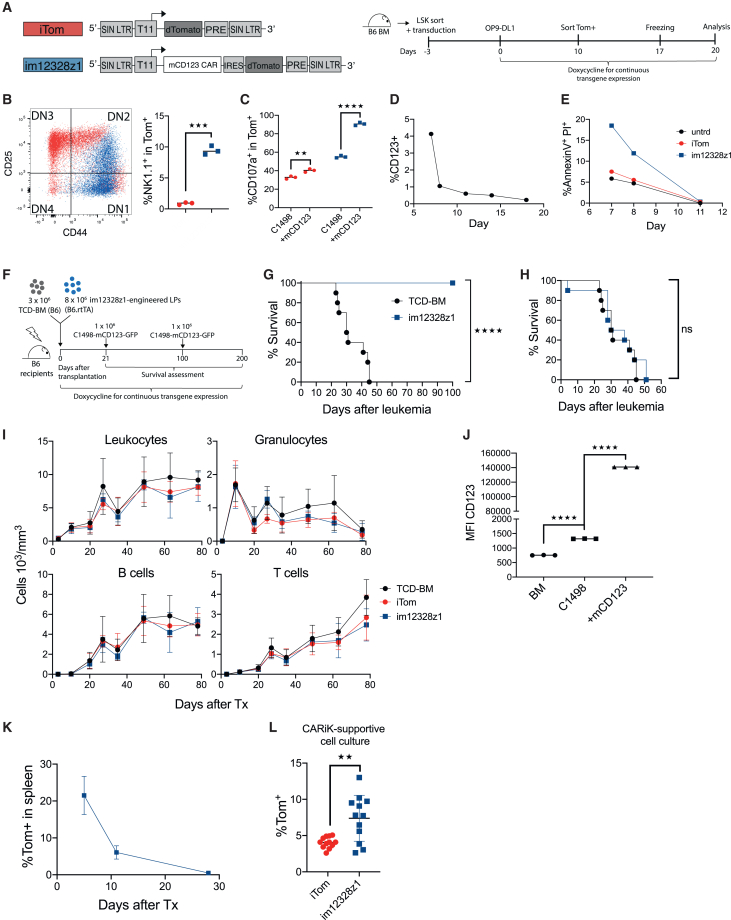
im12328z1-engineered HSPCs differentiate into CARiK cells *in vitro* and provide strong anti-leukemia effects in HCT recipients (A) Representation of the lentiviral vectors: inducible dTomato reporter gene only (iTom) and im12328z1. (B–E) Murine LSK cells were transduced with either im12328z1 or the iTom control vector and consecutively differentiated on OP9-DL1 stromal cells. Flow cytometry analysis was performed within the Tom^+^ gate on day 20 of co-culture. Representative results from one of three independent experiments are shown. (B) Representative flow cytometry plot for CD44 and CD25 expression of im12328z1-engineered lymphoid progenitors (blue) and respective iTom controls (red, left) and frequencies of NK1.1^+^ cells (right). DN, double negative. (C) Responses of engineered lymphoid progenitors upon stimulation with C1498/C1498_mCD123 target cells were quantified by degranulation. (D) CD123 expression on engineered lymphoid progenitors and (E) AnnexinV/PI positivity were measured at indicated time points. (F and G) Irradiated female B6 mice were reconstituted with 3 × 10^6^ B6 TCD-BM cells (*n* = 10/group) and co-transplanted with 8 × 10^6^ B6 im12328z1-engineered lymphoid progenitors. Studies were performed under permanent administration of Dox-containing water or food for transgene expression. Recipients were intravenously challenged with 1 × 10^6^ C1498-mCD123 cells on day 21 after transplantation. Survival was monitored. Results from two independent transplantations were pooled. (H) Survivors were re-challenged with 1 × 10^6^ C1498-mCD123 cells on day 100 and assessed for survival. TCD-BM-only recipients served as controls. (I) Myeloid and lymphoid recovery in irradiated female B6 recipients of 3 × 10^6^ TCD-BM cells (*n* = 4/group) with or without 8 × 10^6^ syngeneic iTom/im12328z1-engineered lymphoid progenitors was graphed. At indicated time points, blood counts were measured and peripheral blood was analyzed for CD45 (total white blood cells), Gr.1 (granulocytes), CD19 (B cells), and CD3 (T cells) by flow cytometry. Experiments were performed once. (J) Primary murine BM, C1498, and C1498-mCD123 cells were comparatively assessed for CD123 expression strength by flow cytometry. Respective results from one of three independent experiments are shown. (K) Frequencies of Tom^+^ progeny in the spleen of im12328z1 transplant recipients over time by flow cytometry analysis (*n* = 3 mice/time point). Experiments were performed once. (L) Splenocytes of recipients of either im12328z1-engineered lymphoid progenitors or respective iTom controls were harvested on day 12 and re-cultured *ex vivo* under CARiK-optimized conditions (*n* = 12). Data are shown as mean ± SEM. Student’s t test (two tailed) and one-way ANOVA with Tukey’s *post hoc* test were used for analysis. Each data point represents an individual sample. Survival curves were compared using Mantel-Cox (log rank) test. ∗*p* < 0.05, ∗∗*p* < 0.01, ∗∗∗*p* < 0.001, ∗∗∗∗*p* < 0.0001.

Lymphoid progenitors were generated using the OP9-DL1 co-culture system after transduction of LSK cells (Lin^−^/Sca1^+^/cKit^+^) with either the im12328z1 CAR or the iTom control vector (Figure 3A).^27^ Despite minimal tonic signaling activity, im12328z1-transduced lymphoid progenitors started to differentiate into a CAR-induced NK-like phenotype as reflected by a maturation block at the double-negative 2 (DN2) T cell progenitor stage (CD4^−^CD8^−^CD25^mid^CD44^+^) and the appearance of increased frequencies of NK1.1^+^ cells (Figure 3B). *In vitro* stimulation of im12328z1-expressing lymphoid progenitors with mCD123 target cells resulted in antigen-specific degranulation (Figure 3C). Due to CD123 expression on im12328z1 CAR-engineered early hematopoietic progenitor cells, increased AnnexinV/PI positivity in im12328z1-transduced progenitors in comparison to negative controls suggested some degree of fratricide. The degree of fratricide was low and eventually lost upon further differentiation and loss of CD123 expression (Figures 3D and 3E). Next, irradiated syngeneic C57BL/6 (B6) recipients were transplanted with 3 × 10^6^ T cell-depleted bone marrow (TCD-BM) cells alone or repleted with 8 × 10^6^ im12328z1-engineered lymphoid progenitors and challenged with a lethal dose of mCD123^+^ leukemic cells (C1498-mCD123-GFP) on day 21 after HCT to assess *in vivo* activity (Figure 3F). The *in vivo* progeny of the im12328z1-engineered progenitor cells mediated up to 100% leukemia-free survival (Figure 3G). A second leukemia challenge of 100-day survivors, however, resulted in a 100% lethality rate, suggesting the absence of long-term activity and memory cell formation (Figure 3H). Since CD123 is expressed during hematopoiesis, we expected graft failure or at least delayed myeloid recovery in HCT recipients. Surprisingly, this was not the case (Figure 3I). Comparison of CD123 on leukemic and bone marrow (BM) cells revealed a 2 log higher expression of CD123 on the C1498_mCD123 cells (Figure 3J). We assume that affinity and avidity specifics of the respective binder that was used to generate our anti-murine CD123 CAR impacted this balance between on-target/off tumor and on-target/on-leukemia effects. *In vivo* detection of the im12328z1-derived progeny was limited and lost by day 30, possibly allowing myeloid recovery to occur (Figure 3K). Next, im12328z1-expressing progeny were isolated on day 12 after HCT from the recipients and maintained either under CARiK-supporting (high IL-2 doses; 1,000 U/mL) or T cell-supporting culture conditions. This resulted in superior proliferation under CARiK-supporting conditions (Figure 3L). In summary, we demonstrate CAR-induced NK cell-like development using a newly generated CAR against CD123 in a murine model for AML.

### *Bcl11b* knockout in CAR-engineered lymphoid progenitors alters the cytokine release profile and cytotoxic responsiveness after specific stimulation *in vitro*

**Figure 4:**
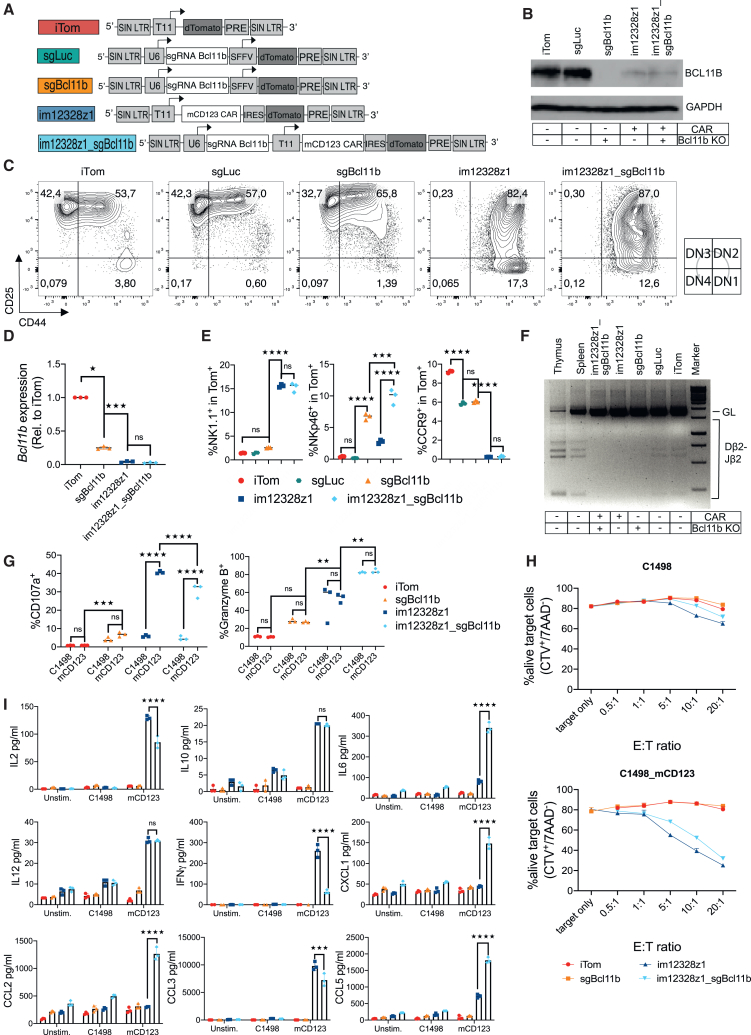
*Bcl11b* knockout in CAR-engineered lymphoid progenitors alters the cytokine release profile and cytotoxic responsiveness after specific stimulation *in vitro* (A) The lentiviral vectors iTom, luciferase-targeted sgRNA for control (sgLuc), Bcl11b-targeted sgRNA (sgBcl11b), im12328z1, im12328z1_sgBcl11b (im12328z1 + sgBcl11b). All constructs are equipped with a dTomato reporter gene. (B–I) Murine LSK cells were transduced with lentiviral vectors and consecutively differentiated on OP9-DL1 stromal cells. Doxycycline was added during the entire culture period from day 1 onward. Cells were sorted for Tom^+^ on day 10 of culture. Flow cytometry analysis was performed within the Tom^+^ gate on day 20 of co-culture. Representative results from one of two independent experiments are shown. (B) Western blot analysis of BCL11B in lysates from bulk cultures of transduced lymphoid progenitor cells. (C) Representative flow cytometry plots of CD44 and D25 expression on *in vitro*-generated engineered lymphoid progenitors. DN, double negative. (D) qPCR analysis of *Bcl11b* expression in transduced lymphoid progenitors. (E) Engineered lymphoid progenitors were assessed for the expression of NK1.1, NKp46, and CCR9 by flow cytometry. (F) Recombination of D and J regions of the TCR-β locus in engineered lymphoid progenitors. Genomic DNA of engineered progenitors was isolated on day 20 of culture and rearrangements were detected by PCR. Splenocytes and thymocytes from WT B6 mice as well as non-transduced lymphoid progenitors were used as controls. GL, germ line. (G) Antigen specificity and functionality of engineered lymphoid progenitors upon stimulation were quantified via granzyme B and CD107a degranulation. (H) Specific cytotoxic activity of engineered lymphoid progenitor cells against either C1498-mCD123 or C1498 control cells using a flow cytometry-based assay. (I) Multiplex cytokine/chemokine analysis of supernatant from engineered lymphoid progenitors that had been co-cultured with target cells for 24 h. A set of 24 different cytokines and chemokines were randomly measured. Data from one experiment are shown as mean ± SEM. Statistics were performed using one-way ANOVA with Tukey’s *post hoc* test. Each data point represents an individual sample. ∗*p* < 0.05; ∗∗*p* < 0.01; ∗∗∗*p* < 0.001; ∗∗∗∗*p* < 0.0001; ns, not significant.

To potentially make CARiK development independent of CAR activation, a *Bcl11b*-targeted sgRNA was added to the construct to allow for CRISPR-Cas9-mediated *Bcl11b* disruption (Figure 4A). Three previously published *Bcl11b*-targeted sgRNAs^19^^,^^32^ were screened for the most effective BCL11B protein reduction (Figure S3; Table S1). CAR transduction of murine LSK cells without *Bcl11b* editing suppressed BCL11B protein production incompletely (Figure 4B). Isolated CRISPR-Cas9-based *Bcl11b* disruption led to a more profound reduction of the protein. Residual BCL11B detection in double-engineered cells might be explained by a proliferative benefit of residual non-transduced cells in culture after the sort. Unexpectedly, early *Bcl11b* disruption alone (sgBcl11b) did not result in a developmental arrest in DN2 (Figure 4C). In contrast, CAR expression blocked differentiation at the DN2 stage at similar levels with or without concomitant *Bcl11b* editing. Concordantly to the data obtained from western blotting, *Bcl11b* expression was significantly decreased by *Bcl11b* disruption and CAR transduction in lymphoid progenitors, as shown by reverse-transcription qPCR (Figure 4D). CAR expression alone drove the development of an NK cell-like phenotype as shown by higher NK1.1^+^ cell frequencies and modest upregulation of NKp46. Increased frequencies of NKp46^+^ cells were observed in the *Bcl11b*-editing-only group. Finally, CRISPR-induced *Bcl11b* knockout in CAR-engineered LSK cells resulted in similar levels of NK1.1^+^ cells; however, NKp46^+^ cells were significantly increased when compared to CAR-only transduced progenitors (Figures 4E and S4A). Of note, expression of the C-C chemokine 9 (CCR9), important for thymic homing,^33^ was completely suppressed in both CAR-expressing constructs. All three, sgBcl11b, im12328z1, and im12328z1_sgBcl11b-engineered lymphoid progenitors completely lacked diversity-joining (D-J) recombination segments within the *Tcrb* locus (Figure 4F). *In vitro* stimulation of im12328z1-engineered lymphoid progenitors with mCD123-expressing C1498 target cells already resulted in specific activation as shown by granzyme B release and CD107a degranulation. However, perforin production was minimal or not detected. Of note, the *Bcl11b* knockout alone resulted in some level of non-specific activation, resembling NK cell-like antigen-independent killing behavior (Figures 4G, S4B, and S4C). For comparative functional assessment of the differently engineered progenitors at this early stage of development, these cells were tested in an *in vitro* killing assay and randomly screened using a cytokine multiplex assay. Additional *Bcl11b* knockout in CAR-engineered lymphoid progenitors did not alter cytotoxic responsiveness to antigen-expressing target cells (Figure 4H); however, it resulted in a shifted chemokine/cytokine release profile for seven out of 24 screened chemokines and cytokines. IL-10 and IL-12 were comparatively excreted upon stimulation with or without *Bcl11b* knockout, albeit significantly more when compared to the other controls (Figure 4I). Taken together, *Bcl11b* disruption alone is no substitute for CAR activation-dependent NK-directed lymphoid development, suggesting a more ambiguous role of BCL11B during lymphoid progenitor cell differentiation. Importantly, *Bcl11b* knockout in CAR-engineered lymphoid progenitors resulted in a higher expression of the NK marker NKp46. This was associated with an altered antigen-specific cytotoxic activity and a distinct cytokine/chemokine release profile *in vitro*.

### CAST-seq reveals a favorable safety profile for *Bcl11b* knockout

CRISPR-Cas9 genome editing carries the risk of genotoxicity caused by off-target effects. Therefore, we searched for potential structural variations induced by the *Bcl11b*-targeted knockout. Chromosomal aberration analysis by single-targeted linker-mediated PCR sequencing (CAST-seq) was performed to identify and quantify gross chromosomal rearrangements derived from on-target and off-target activities of the Cas9 nucleases. CAST-seq confirmed a high degree of specificity of the *Bcl11b*-targeting nuclease. No off-target-mediated translocation events were identified (Figures S5A and S5B). At the on-target site, CAST-seq revealed the presence of rare large deletions of up to 3 kb at the *Bcl11b* locus. Short-fragment PCR amplification of the on-target site indicated medium-size deletions to be causative for the *Bcl11b* knockout (Figures S5C and S5D). ICE analyses of Sanger sequencing (https://doi.org/10.1089/crispr.2021.0113) showed that up to 96% of the alleles were modified, with deletions of 34 or 35 bp being the most frequent (Figure S5E). These data collectively suggest the specificity of CRISPR-Cas9-mediated *Bcl11b* targeting.

### Potential CAR gene-driven mutagenesis of CAR-modified lymphoid progenitors

**Figure 5:**
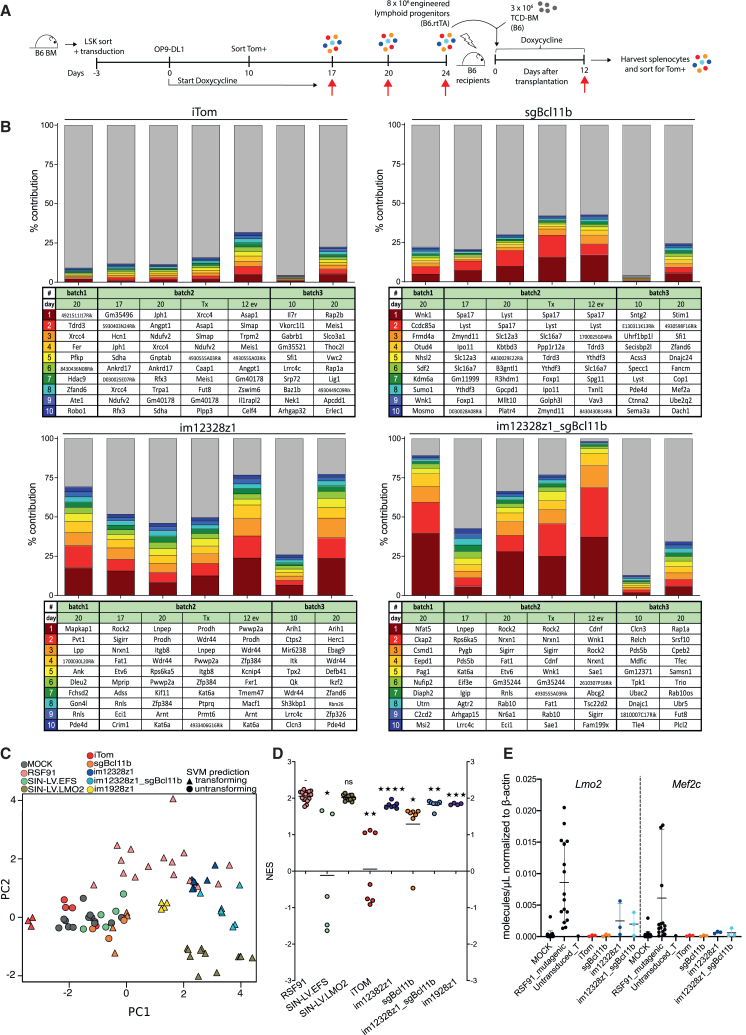
Potential CAR gene-driven mutagenesis of CAR-modified lymphoid progenitors (A) Murine LSK cells were transduced with sgBcl11b, im12328z1, im12328z1_sgBcl11b, or the iTom control vector and differentiated into lymphoid progenitors using the OP9-DL1 co-culture system. ISA was performed during *in vitro* differentiation on days 17, 20, and immediately before co-transplantation on day 24 (*n* = 3 independent *in vitro* cultures). Irradiated female B6 recipients were reconstituted with 3 × 10^6^ B6 TCD-BM and co-transplanted with 8 × 10^6^ engineered lymphoid progenitors (*n* = 3–5 mice/group). Studies were performed under permanent administration of Dox-containing water or food for transgene expression. Progeny cells were retrieved from the recipients and assessed for clonal evolution 12 days after transfer. (B) The top 10 integration sites at the different time points. Data of three independent cultures are shown. Each colored bar represents a unique insertion site from the top 10 most abundant sequences. The gray bars represent all other background insertions. All shown insertion sites correspond to a unique chromosomal position. (C) PCA of the four gene-edited lymphoid progenitor groups on day 20 of culture. Data of 4–7 independent cultures are shown. They were compared to sorted DN2 cells either non-transduced (mock) or transduced with genotoxic vectors. The support vector machine (SVM) predictions are represented with different shapes (circle, non-transforming; triangle, transforming) and the vector designs with color codes (MOCK, gray; SIN-LV.EFS, green; RSF91, red; SIN_LV.LMO2, dark gray). (D) NES values obtained with SAGA-XL-GSEA using the DN2 features. The four constructs were compared to metadata of DN2-sorted cells. (E) *Lmo2* and *Mef2* mRNA levels measured by ddPCR in gene-edited lymphoid progenitors and respective controls on day 20 of culture. All values were normalized to the expression of the housekeeping gene *β-actin*. Each data point represents an individual sample. Data are shown as mean ± SEM.

BCL11B is a transcription factor that acts as a haploinsufficient tumor suppressor.^34^ Mutations and deletions in the human ortholog BCL11B have been identified in human T cell acute lymphoblastic leukemia (T-ALL)^34^ and in murine lymphomas.^35^ To evaluate for potential clonal dominance, we conducted a comprehensive DNA insertion-site analysis (ISA) over time (Figure 5A). For ISA, we amplified vector-genome junctions using the integration-site pipeline for paired-end reads (INSPIIRED) workflow.^36^ ISA was performed during *in vitro* differentiation and after co-transplantation into BM recipients on day 12. Mean vector copy numbers (mVCNs) per cell were comparable between groups (Figure S6A). Day 17 polyclonality was followed by a reduction of unique insertion sites and sequence pool diversity indicating a certain degree of clonal restriction over time, comparable for all vector types (Figures S6B and S6C). To further characterize the insertional patterns, we identified the top 10 integration sites over time. INSPIIRED^36^ did not indicate vector configuration-driven insertional mutagenesis, or clonal selection *in vivo* (Figure 5B). Normalizing the abundance values of each insertion site for the number of transduced cells showed that seemingly dominant clones in im12328z1 and im12328z1_sgBcl11b contributed only slightly to overall hematopoiesis (Figure S6D). Importantly, no high-risk insertions close to proto-oncogenes were detected (Figure 5B). Comparison of the exact chromosomal location of insertions within progenitors that were derived from independent cultures revealed no overlap of insertion sites among all batches, suggesting a semi-random vector integration for all constructs (Figure 5B). These data argue against a systemic selection pressure by insertional mutagenesis at the respective mVCN levels per cell. However, the risk of insertional transformation can be linked to vector design, integration site profiles, mean vector copy number, and the transgene itself. Since the transformation of healthy murine hematopoietic progenitors to immortalized mutants was shown to correlate with a specific oncogenic gene expression signature,^37^ we used a novel version of the surrogate assay of genotoxicity assessment (SAGA) that was specifically adapted to detect lymphoid mutants.^38^ Microarray analyses of RNA samples were performed to investigate whether lymphoid progenitors that were arrested in DN2 were associated with a particular oncogenic signature. We analyzed separate batches of DN2-sorted samples after 20 days of *in vitro* differentiation. Gene expression was compared with the expression profile of murine HSPCs that had been transduced with genotoxic positive control vectors (RSF91 and SIN-LV.LMO2), as previously published.^38^ Principal-component analysis (PCA) revealed a distinct clustering of all four lymphoid progenitor populations within the gene expression landscape of DN2-sorted cells (Figure 5C). iTom and sgBcl11b-edited progenitor cells clustered with the negative controls. In contrast, PCA showed a clear separation of im123228z1 and im12328z1_sgBcl11b cells that rather clustered in proximity of transforming vectors. We used the lymphoid features found on DN2-sorted samples for gene set enrichment analysis (GSEA) and compared each sample to the mean of mock controls. For both CAR-transduced samples, the mean normalized enrichment score (NES) >1 indicated an enrichment of the lymphoid genotoxicity predictors and, therefore, a potential mutagenic risk (Figure 5D). Using a murine CAR against CD19 as control in the respective progenitors, we showed that potential CAR-driven transformation might be driven by the CAR independently of its antigen specificity (Figures 5C and 5D). Of note, neither im123228z1- nor im12328z1_sgBcl11b-transduced lymphoid progenitors highly overexpressed *Lmo2* or *Mef2c*, two pivotal proto-oncogenes that have been shown to be dysregulated in DN2-arrested progenitors^38^ (Figure 5E). However, this needs to be regarded with great caution since a certain elevation of *Lmo2* expression was observed. In summary, these data suggest that both im12328z1 and im12328z1_sgBcl11b inserts carry a certain mutagenic risk. This does not seem to be caused by the vector configuration but rather by the CAR expression itself.

### Combining CAR engineering with CRISPR-Cas9-induced *Bcl11b* knockout enhances NK cell-like properties and prolongs anti-leukemic efficacy *in vivo*

**Figure 6:**
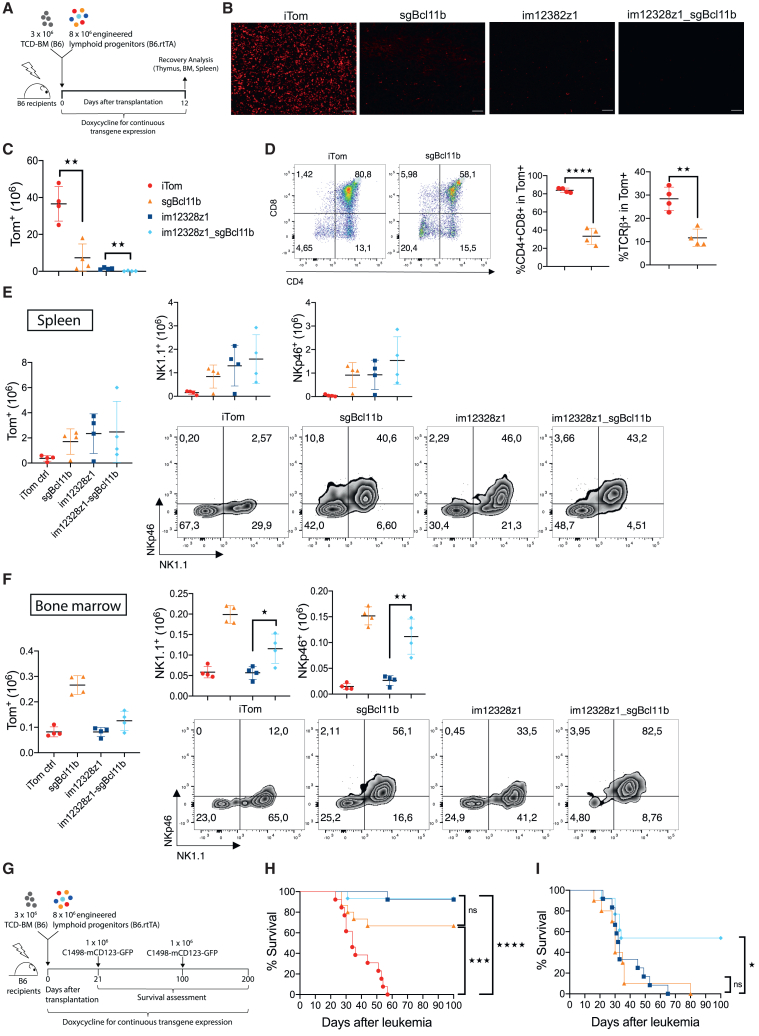
Combining CAR engineering with CRISPR-Cas9-induced *Bcl11b* knockout enhances NK cell-like properties and prolongs anti-leukemic efficacy *in vivo* (A) Irradiated female B6 recipients were reconstituted with 3 × 10^6^ B6 TCD-BM and co-transplanted with 8 × 10^6^ engineered lymphoid progenitors (*n* = 4 mice/group). Studies were performed under permanent administration of Dox-containing water or food for transgene expression. (B) Thymic sections were imaged for Tom^+^ cells. Original magnification, ×20. (C) Cells from harvested thymi were analyzed by flow cytometry for Tom^+^ progeny of co-transplanted lymphoid progenitors. (D) The progeny of lymphoid progenitors were retrieved from thymus on day 12 after transfer. Representative flow cytometry plots show CD4/CD8 expression (left) and frequencies of TCR-β^+^ and CD4^+^CD8^+^ progeny within the Tom^+^ gate (right). Respective analysis of the spleen (E) and BM (F) are depicted as absolute numbers of Tom^+^, NK1.1^+^, and NKp46^+^ cells. Experiments were performed once. (G and H) Irradiated female B6 recipients of 3 × 10^6^ TCD-BM and 8 × 10^6^ syngeneic B6 engineered lymphoid progenitors (*n* = 13–15 mice/group) were challenged with 1 × 10^6^ C1498-mCD123 leukemia cells on day 21 after transplantation and consecutively monitored for survival. Results from three independent transplantations were pooled. (I) Survivors were re-challenged with 1 × 10^6^ C1498-mCD123 cells on day 100. Data from two independent experiments were pooled (*n* = 10–13 mice/group). Student’s t test (two tailed) and one-way ANOVA with Tukey’s *post hoc* test were used for analysis. Survival curves were compared using Mantel-Cox (log rank) test. Data are shown as mean ± SEM. Each data point represents an individual sample. ∗*p* < 0.05; ∗∗*p* < 0.01; ∗∗∗*p* < 0.001; ∗∗∗∗*p* < 0.0001; ns, not significant.

To track the development of *Bcl11b*-edited CAR-expressing lymphoid progenitor cells *in vivo*, irradiated syngeneic C57BL/6 (B6) recipients were transplanted with 3 × 10^6^ TCD-BM cells, which were enriched with 8 × 10^6^ engineered lymphoid progenitors (Figure 6A). Although co-transplanted iTom control lymphoid progenitors repopulated the thymus by day 12 after transplantation, the progeny of im12328z1 and im12328z1_sgBcl11b cells failed to do so (Figures 6B and 6C) and were predominately identified in BM and spleen (Figures 6E and 6F). sgBcl11b progenitors, however, maintained some thymic immigration capacity, albeit with reduced expression of T cell markers (Figure 6D). Flow cytometry analysis of spleen and BM-derived progeny demonstrated that CARiK development can be further increased by additional *Bcl11b* editing (Figures 6E, 6F, and S7). We next studied the anti-leukemia potential of double-edited lymphoid progenitors. Respective recipients were challenged after 21 days with a lethal dose of mCD123^+^ leukemic cells (Figure 6G). After co-transplantation, im12328z1_sgBcl11b CARiK cells demonstrated potent anti-leukemic activity (Figure 6H). Of note, the progeny of sgBcl11b cells also led to significant anti-leukemic effects suggesting the *in vivo* occurrence of an effector cell population with CAR-independent functionality. In order to assess the durability of anti-leukemic activity, a second leukemia challenge of day-100 survivors was conducted. The observed 100% lethality rate of im12328z1 and sgBcl11b recipients confirms the absence of long-term activity or functional memory cell formation (Figure 6I). Double-edited im12328z1_sgBcl11b cells, however, prolonged the anti-leukemic activity with up to 54% mice surviving after re-challenge. These data suggest that the combination of CAR transduction and *Bcl11b* knockout gives rise to a distinct cell population with improved anti-leukemic potency.

### The *in vivo* progeny of im12328z_sgBcl11b lymphoid progenitors shows a shift toward transcriptional activation associated with NK cell identity and cytotoxicity

**Figure 7:**
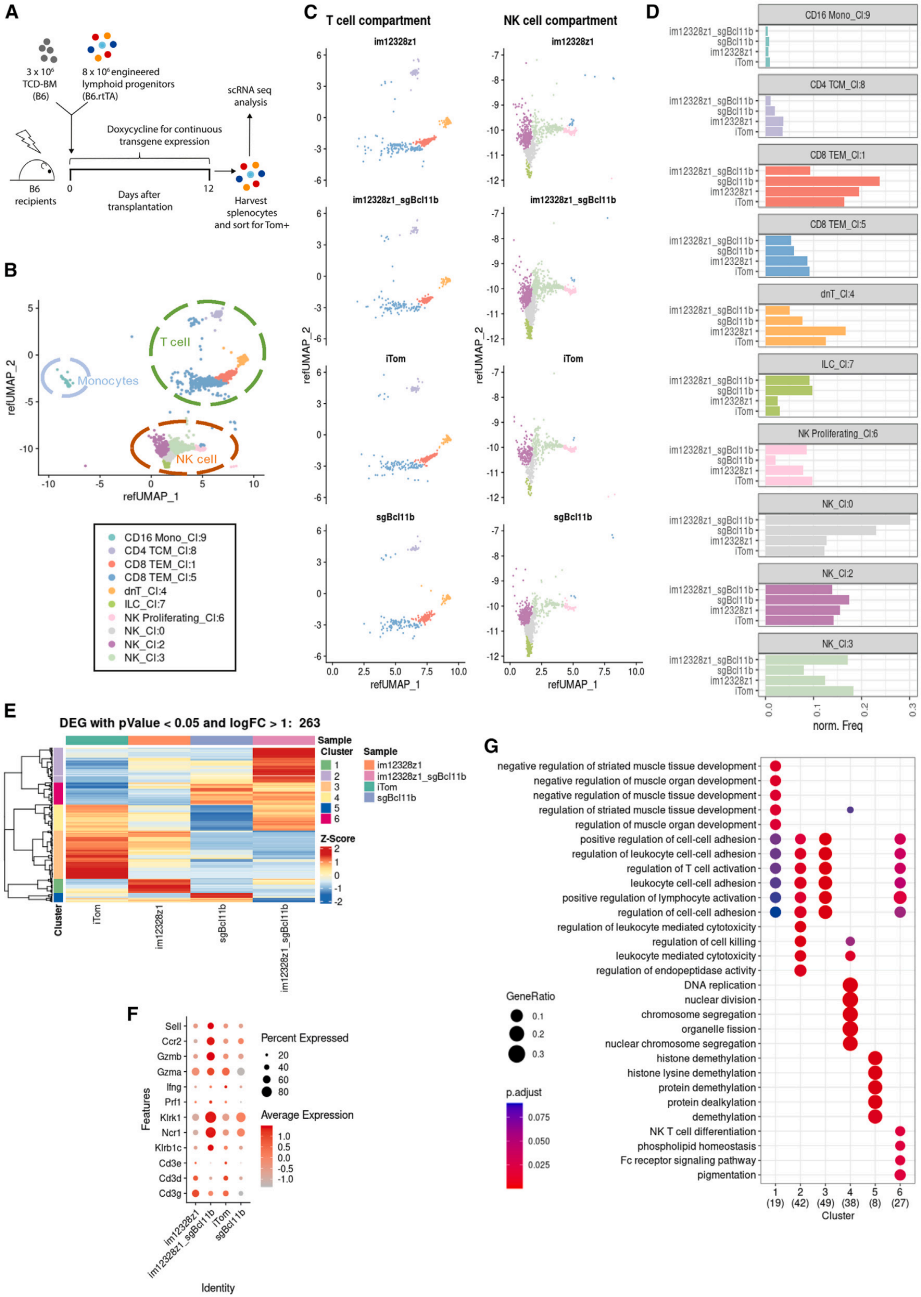
The *in vivo* progeny of im12328z_sgBcl11b lymphoid progenitors shows a shift toward transcriptional activation associated with NK cell identity and cytotoxicity (A) Irradiated B6 recipients were reconstituted with 3 × 10^6^ B6 TCD-BM cells and co-transplanted with 8 × 10^6^ engineered lymphoid progenitors (*n* = 5 mice/group). Studies were performed under permanent administration of Dox-containing water or food for continuous induction of transgene expression. scRNA-seq analysis was accomplished on spleen-derived progeny of gene-edited lymphoid progenitors 12 days after further *in vivo* differentiation. (B) UMAP visualization of scRNA-seq data displaying cell types identified from isolated lymphocytes across all samples. Integrated datasets (*n* = 7,715 cells) reveal T cells (green circle), NK cells (red circle), and monocytes (light blue circle) as major cell types. Each major cell type is further subdivided into smaller subtypes, classified based on a combination of cell type and gene expression clusters (UMAP clusters Cl). (C) Dimensional reduction plot for each sample, focusing on T and NK cells. The integrated dataset was split into individual samples (im12328z1, im12328z1_sgBcl11b, iTom, sgBcl11b) to visualize the cell type plasticity introduced by cell engineering. (D) Differently engineered cells exhibit a distinct cell type profile. Bar plots for each cell subtype display normalized cell frequencies for each sample. (E) Clustered heatmap visualizing distinct expression profiles of differently engineered samples. Genes selected for the heatmap show differential expression compared to the iTom control dataset (absolute log 2 fold change [log2FC] >1, FDR < 0.05). The heatmap displays *Z*-scored average gene expression of 263 selected genes, with hierarchical clustering isolating six clusters (hierarchical clusters) with similar expression profiles. (F) Expression of selected marker genes. The dot plot shows the expression levels of specific marker genes across all cells within each sample. T cell markers: Cd3g, Cd3d, Cd3e. NK cell markers: Ncr1, Klrb1c, Klrk1. Cytotoxicity markers: Prf1, Ifng, Gzma, Gzmb. Activation/homing markers: Sell, Ccr2. Each row represents a marker gene, while each column corresponds to a distinct sample. Dot size indicates the proportion of cells in each sample expressing the respective marker gene, and color intensity reflects the average expression level of that gene. This figure highlights the differential expression of key marker genes across all samples, providing insights into the impact of different cellular modifications. (G) Gene signatures for regulation of cell killing and NK T cell differentiation are enriched in double-edited samples (im12328z1_sgBcl11b). GSEA was conducted on clusters identified by hierarchical clustering using the Gene Ontology (GO) category "Biological Process." The top five significant categories for each cluster are plotted, with dot size representing the gene ratio (number of genes detected in each cluster divided by the total number of genes in each category). The p.adjust values indicate the significance of enrichment.

Using single-cell RNA sequencing (scRNA-seq) technology, we aimed to further characterize the *in vivo* progeny of the differently engineered lymphoid progenitor cells (Figure 7A). Uniform manifold approximation and projection (UMAP) visualization of scRNA-seq data grouped 10 cell-type clusters (termed UMAP clusters) across all samples (Figures 7B and S8B). Lacking a respective murine dataset, reference-based cell-type mapping was done using a CITE-seq dataset of human PBMCs. The distribution of mapping scores (Figure S8A) showed the human dataset to be representative for murine cells. Integrated datasets grouped the 10 UMAP clusters into three meta-clusters, namely T cells, NK cells, and monocytes (Figure 7B). The progeny of double-edited im12328z1_sgBcl11b progenitor cells grouped most frequently among NK cluster 0 (NK_Cl:0, gray color). This suggests that additional *Bcl11b* deletion in CAR-engineered lymphoid progenitors leads to a differentiation shift within the NK meta-cluster (Figures 7C and 7D). Hierarchical clustering of the 263 most variably expressed genes within the progeny of the differently engineered lymphoid progenitors further illustrated their individual identity (Figure 7E). Within those 263 genes, six hierarchical clusters (different from UMAP clusters) were identified. The hierarchical cluster #2 within the im12328z1_sgBcl11b population was clearly distinct from the three other groups (Figure 7E). Furthermore, differential expression of key marker genes in the im12328z1_sgBcl11b progeny indicated accentuated NK cell-like differentiation and increased cytotoxicity in comparison to the im12328z1-only edited progeny (Figure 7F). This pronounced expression profile was further associated with increased transcriptional activity in the functional context of regulation of cell killing and NK T cell differentiation (Figure 7G). In summary, the progeny of im12328z_sgBcl11b-double-edited lymphoid progenitors showed a prominent shift toward transcriptional activation associated with NK cell identity and cytotoxicity.

## Discussion

Redirecting the differentiation of peripheral blood T cells to acquire phenotypic NK cell markers has become an exciting field of cellular therapy. These mostly CD8^+^ T cells, termed innate-like, make them candidates for cellular immunotherapeutic approaches due to dual-target recognition and have demonstrated anti-tumor activity in humanized mouse models.^39^^,^^40^^,^^41^

Here, we show that a combination of *Bcl11b* editing and CAR activation can be used to foster CARiK development with prolonged anti-leukemic functionality—a novel CAR-based NK-like product from hematopoietic progenitor cells that can mediate strong and more durable anti-leukemic effects. Our findings are clinically relevant since this strategy represents a novel platform using CAR effectors with unique NK-like properties.

BCL11A (CTIP1) and BCL11B (CTIP2), two related C2H2 zinc-finger protein transcription factors, were originally described by Avram and colleagues.^5^^,^^6^ As alpha/beta T cell development was blocked at the level of CD4^−^/CD8^−^ double-negative thymocyte development in BCL11B-deficient mice, the importance of BCL11B in the differentiation and survival of thymocytes started to be recognized.^7^^,^^8^^,^^9^^,^^10^^,^^11^^,^^12^^,^^14^ We observed earlier that CAR expression during early lymphoid differentiation gave rise to a lymphoid cell type with NK-like features, which we termed CARiK cells. A key observation was that CAR-mediated signaling led to a downregulation of BCL11B, this important transcriptional gatekeeper of T- and NK cell development. However, signaling via the CAR was a prerequisite for this phenomenon, since signaling-deficient controls developed into normal T cells. Signaling initiated by TCR activation on thymocytes has been reported to direct reprogramming of the gene-expression network in progenitor cells that drives T cell differentiation and mitogen-activated protein (MAP) kinase pathways have been identified to dynamically regulate posttranscriptional modifications of BCL11B, thereby modifying its activity on its transcriptional target.^42^ Continuous antigen stimulation or significant tonic signaling activity of a CAR leads to chronic cell activation.^43^ Of potential relevance, prolonged signaling via CD3ζ results in BCL11B ubiquitination and degradation.^42^

Ueda and colleagues observed a similar phenomenon when CAR-transduced human iPSCs were used to generate human “T cells” *in vitro*. They reported this NK-like shift to occur only when a CD28-costimulated CAR was used, while “normal” T cell development took place upon 4-1BB co-stimulation.^44^ We and others have tried to reproduce this in a murine model; however, 4-1BB in the murine system has proved to be of little functional relevance.^45^ Since BCL11B directly or indirectly disables an essential switch of the T-lineage-specific commitment, we added a sgRNA directed against *Bcl11b* with the intention to support more CAR-independent NK cell-like development. Together with the constitutively expressed CRISPR-Cas9 nuclease, this sgRNA resulted in highly efficient knockout of the target gene. We hypothesized that CRISPR-mediated deletion of *Bcl11b* would finally substitute for chronic signaling effects of a CAR. This was not the case, since controls with *Bcl11b* deletion only showed a distinct differentiation and functionality pattern *in vitro* and *in vivo*. In contrast, double-engineered progenitors seemingly differentiated very similarly throughout the DN stages and had practically identical short-term anti-leukemia effects *in vivo* to CAR-only-transduced progenitors. However, upon stimulation, they displayed a distinct cytokine release profile. This included IL-2, IL-6, interferon (IFN)-γ, CXCL1, CCL2, CCL3, and CCL5. The mechanistic context of this observation remains unclear; however, it seems to be associated with prolonged functionality of the respective progeny *in vivo*, which was a distinctive feature of the im12328z1_sgBcl11b CARiK cells. In this context, one is tempted to hypothesize that dual targeting via the CAR and the cytotoxic NK cell-like machinery might result in different degrees of exhaustion as shown earlier in a model with bi-reactive T cells.^46^ Performing scRNA-seq analysis on the *in vivo* progeny of im12328z1_sgBcl11b cells revealed an increased cytokine activation profile in the identity-plot analysis when compared to the other groups. However, this reflects one specific time point after transfer and respective transcriptional activity might have been missed before or thereafter.

Perforin is essential for the lytic function of lymphocytes^47^^,^^48^ and is necessary for delivery of granzyme B to target cells.^49^^,^^50^ In our system, antigen-specific stimulation of the lymphoid progenitors did not lead to detectable perforin production. Somewhat unexpectedly, we observed CD107a appearance on the cell surface and granzyme B was released, the latter as well independently of antigen-specific stimulation via CD123. Blotting granzyme B against CD107a did show both granzyme B and CD107 degranulation, but mostly not simultaneously, suggesting separate activities.^51^

After final differentiation and “education,” NK cells mediate cytotoxic effects via two different mechanisms: granzyme-based killing and death receptor-mediated apoptosis.^52^ Granzyme B and CD95 ligand are thereby localized in distinct cellular compartments.^53^^,^^54^ Prager and colleagues have shown that CD95L expression was confined to CD107a-degranulating NK cells.^51^ While only cells that released their lytic granules also mobilized CD95L-containing vesicles, the degranulation of these vesicles seems to be differently regulated.^55^ Since perforin production has been shown to be a prerequisite for granzyme-mediated killing, one might hypothesize that specific killing at this early stage of differentiation might be driven by CD95L-dependent mechanisms. Apart from this, the transcription factor BCL11B has been reported to reduce antigen-dependent clonal expansion and cytolytic activity was altered with decreased granzyme B and perforin.^21^ Defects in LAMP1/CD107a resulted in retention of perforin in the transport vesicles instead of delivering it to the lytic granules.^56^

Nevertheless both im12328z1 and im12328z1-sgBcl11b cells mediated some degree of specific cytotoxic activity already at this stage of differentiation, and a possible dissociation of antigen-specific granzyme B and perforin production did not abolish antigen-specific responses as measured by CD107a.

*Bcl11b* binding across the genome demonstrates distinct cell-type-specific motif preferences in comparative studies using murine pro-T cells and innate lymphoid cells.^19^ Elegant studies in VaV-iCre and the Lck-Cre models showed that the effects of *Bcl11b* deletion before commitment were more severe than those after commitment. The most time-sensitive genes included *CD3g*, *CD3d*, and *CD3c* among the *Bc11b*-dependent genes, and *CD7* among genes that were repressed by *Bcl11b*.^32^ However, BCL11B expression was recently reported to increase during human NK cell differentiation.^24^ Although this generated strong evidence for an important role of *BCL11B* in human NK cell differentiation, recent data have not supported *Bcl11b* as a prerequisite for the differentiation of CD11b-expressing murine NK cells.^24^ Nevertheless, transient expression of *Bcl11b* in immature murine NK cells has been reported.^11^ This might, in part, explain the discrepancies we observed with regard to CD7 expression on engineered UCB-derived progenitors. Despite the fact that canonical NK cell differentiation shows significant differences between humans and mice, *Bcl11b* has been shown to be critical for raising adaptive NK cell responses to cytomegalovirus in humans and mice. In this context, *BCL11B* was most abundantly transcribed in adaptive NK cells acting as a transcriptional activator for differentiation and proliferation.^24^ These findings seem paradoxical and suggest a complex regulatory network wherein the fate of differentiating NK and T cell populations is driven by reciprocal BCL11B-dependent gene programs.

Apart from its central role in lymphoid development, *Bcl11b* is a tumor-suppressor gene, which raises concerns about leukemic transformation associated with its knockout.^34^^,^^35^^,^^57^^,^^58^ In mice, heterozygosity of *Bcl11b* promotes clonal expansion and differentiation arrest in murine thymocytes^30^ in part by an increase of β-catenin, and an acquired loss of *H2AX* was shown to induce blast crisis of chronic myelogenous leukemia.^59^ This suggests that a differentiation arrest triggered by BCL11B deficiency can represent an important step toward tumor development.^60^ In humans, reduced levels of the BCL11B protein are a key event in the multistep progression of adult T cell leukemia and lymphoma (ATLL)^61^ and *BCL11B* was aberrantly expressed in a cohort of T-ALL patients whereby low expression predicted inferior survival.^62^ Human T cell lymphoblastic virus causes ATLL and seems to be linked to allelic loss of *BCL11B* in human and respective mouse models.^61^^,^^63^

Although we could not identify any prominent high-risk insertions in any group, and the clonal distribution within the lymphoid progenitor pool remained comparable to their *in vivo* progeny, transformation can be driven independently of integration sites by the transgene itself. In the light of the current announcement of the US Food and Drug Administration (FDA) to investigate serious risk of T cell malignancies following BCMA- and CD19-directed autologous CAR T cell immunotherapies, this could be of special relevance.^64^^,^^65^ Since the exact mechanism of the CAR-induced differentiation shift remains incompletely understood, further translational development must be planned with great caution. Despite these caveats, our data provide further evidence for the significant plasticity in lymphoid cell development with CAR engineering as a means to foster adaptive NK cell functions and to further enhance NK functionality by targeting *Bcl11b*.

## Materials and methods

### Mice

B6 (H2^b^) mice were purchased from Charles River Laboratories. R26-M2rtTA knockin mice (B6.rtTA, H2^b^) express a reverse tetracycline-controlled transactivator for doxycycline (Dox)-inducible transgene expression. Cas9 knockin mice (B6.CAG-cas9∗-EGFP, H2^b^) with B6 background were a gift from A. Schambach (Hannover Medical School). To create Cas9_R26-M2rtTA knockin mice (B6.CAG-cas9∗-EGFP/rtTA, H2^b^) Cas9 knockin mice (B6.CAG-cas9∗-EGFP, H2^b^) were crossed onto R26-M2rtTA knockin mice (B6.rtTA, H2^b^).

### Cell lines

The murine C1498 myeloid leukemia cell line (H2^b^, B6 origin) was transduced with retroviral supernatant carrying murine CD123 alone in the pAlpha.SIN.MPSV.wPRE vector or linked with an IRES GFP cassette to generate the murine CD123^+^ cell lines C1498-mCD123 or C1498-mCD123-GFP. For isolation of stable mCD123 expression, cells were sorted twice for CD123 or CD123 and GFP (FACSAria Ilu, BD Biosciences). C1498, C1498-mCD123, C1498-mCD123-GFP, and 58α-β cells were cultured in RPMI 1640 and 293T cells were cultured in DMEM (Capricorn). Both media were supplemented with 10% heat-inactivated fetal calf serum (FCS) (Capricorn), l-glutamine, HEPES buffer, and penicillin/streptomycin (all Gibco, Thermo Fisher Scientific). OP9-DL1 cells were cultured in complete αMEM (Life Technologies) supplemented with 20% FCS, l-glutamine, HEPES buffer, and penicillin/streptomycin. The NFAT cell line expressing an NFAT-inducible GFP reporter construct was cultured in DMEM (Capricorn) supplemented with 8% heat-inactivated FCS (Capricorn), l-glutamine, HEPES buffer, and penicillin/streptomycin (all Gibco, Thermo Fisher Scientific). All cell lines were tested for mycoplasm negativity by PCR.

### Generation of antibodies against CD123

The antibody selection was performed as described previously with modifications.^66^ In brief, for panning procedure, the antigen was immobilized on a high-binding 96-well plate (Corning, Costar). 4 μg of recombinant murine CD123 with a human Fc tag (Sino Biological, 50810-M02H) in PBS was coated onto the wells at 4°C overnight. Next, the wells were blocked with 350 μL of 2% MBPST (2% [w/v] milk powder in PBS; 0.05% Tween 20) for 1 h at room temperature (RT)and then washed three times with PBST (PBS; 0.05% Tween 20). Before adding the libraries to the coated wells, the HAL9/10 libraries^31^ (5 × 10^10^ phage particles) were preincubated with 4 μg of an unrelated protein with a human Fc part (blocked also with 2% MPBST) for 1 h at RT, to remove human Fc fragment binders from the libraries. Subsequently, the supernatant was transferred to the antigen-coated wells, incubated for 2 h at RT, and washed 10 times. Bound phage were eluted with 150 μL of trypsin (10 μg/mL) at 37°C for 30 min and used for the next panning round. The eluted phage solution was transferred to a 96-deep-well plate (Greiner Bio-One, Frickenhausen, Germany) and incubated with 150 μL of *Escherichia coli* TG1 (optical density 600 [OD_600_] = 0.5) first for 30 min at 37°C and then for 30 min at 37°C and 650 rpm to infect the phage particles. 1 mL of 2xYT-GA (1.6% [w/v] tryptone, 1% [w/v] yeast extract, 0.5% [w/v] NaCl (pH 7.0), 100 mM D-glucose, 100 μg/mL ampicillin) was added and incubated for 1 h at 37°C and 650 rpm, followed by addition of 1 × 10^10^ colony-forming units (CFU) M13KO7 helper phage. Subsequently, the infected bacteria were incubated for 30 min at 37°C followed by 30 min at 37°C and 650 rpm before centrifugation for 10 min at 3,220 × *g*. The supernatant was discarded and the pellet resuspended in fresh 2xYT-AK (1.6% [w/v] tryptone, 1% [w/v] yeast extract, 0.5% [w/v] NaCl [pH 7.0], 100 μg/mL ampicillin, 50 μg/mL kanamycin). The antibody phages were amplified overnight at 30°C and 650 rpm and used for the next panning round. In total, three panning rounds were performed. In each round, the stringency of the washing procedure was increased (20× in panning round 2, 30× in panning round 3) and the amount of antigen was reduced (2 μg in panning round 2 and 1 μg in panning round 3). After the third panning round, plates containing single clones were used to select monoclonal antibody clones for the screening ELISA.

To screen monoclonal antibody clones, soluble antibody fragments (scFvs) were produced in 96-well polypropylene MTPs (U96 PP, Greiner Bio-One) as described before.^66^ Briefly, 150 μL of 2xYT-GA was inoculated with the bacteria bearing scFv-expressing phagemids. MTPs were incubated overnight at 37°C and 800 rpm in an MTP shaker (Thermoshaker PST-60HL-4, Lab4You, Berlin, Germany). A volume of 180 μL of 2xYT-GA in a PP-MTP well was inoculated with 20 μL of the overnight culture and grown at 37°C and 800 rpm for 90 min (approximate OD_600_ of 0.5). Bacteria were harvested by centrifugation for 10 min at 3,220 × *g* and the supernatant was discarded. To induce expression of the antibody genes, the pellets were resuspended in 200 μL of 2xYT supplemented with 100 μg/mL ampicillin and 50 μM isopropyl-beta-D-thiogalactopyranoside (IPTG) and incubated at 30°C and 800 rpm overnight. Bacteria were pelleted by centrifugation for 20 min at 3,220 × *g* and 4°C.

For the ELISA, 200 ng of antigen was coated on 96-well microtiter plates (high binding, Greiner) in PBS (pH 7.4) overnight at 4°C. As control, 200 ng of a human Fc fusion protein was coated. After coating, the wells were blocked with 2% MPBST for 1 h at RT, followed by three washing steps with H_2_O and 0.05% Tween 20. Supernatants containing secreted monoclonal scFv were mixed with 2% MPBST (1:2) and incubated onto the antigen-coated plates for 1 h at 37°C followed by three H_2_O and 0.05% Tween 20 washing cycles. Bound scFvs were detected using murine mAb 9E10, which recognizes the C-terminal c-myc tag (1:50 diluted in 2% MPBST) and a goat anti-mouse serum conjugated with horseradish peroxidase (HRP) (A0168, Sigma) (1:42,000 dilution in 2% MPBST). Bound antibodies were visualized with tetramethylbenzidine (TMB) substrate (20 parts TMB solution A [30 mM potassium citrate]; 1% [w/v] citric acid [pH 4.1]) and 1 part TMB solution B (10 mM TMB; 10% [v/v] acetone; 90% [v/v] ethanol; 80 mM H_2_O_2_ [30%]) were mixed). After stopping the reaction by addition of 1 N H_2_SO_4_, absorbance at 450 nm with a 620-nm reference was measured in an ELISA plate reader (Epoch, BioTek). Monoclonal binders were sequenced and analyzed using VBASE2 (www.vbase2.org). The binding to cell-bound CD123 was confirmed by flow cytometry.

### Gamma-retroviral/lentiviral constructs and production of supernatant

The murine CD123 CAR construct (im12328z1) contains a phage display-derived anti-CD123 scFv, the transmembrane domain and co-stimulatory domain from mouse CD28, and the CD3ζ signaling domain with one functional ITAM. For the transduction of LSK cells, the CAR sequences were cloned into a lentiviral backbone under the control of the Tet-On T11 promoter followed by IRES dTomato (Tom) cassette. The sgRNA against Bcl11b (sgBcl11b) was previously described^32^ and cloned together with SFFV dTomato into a lentiviral backbone under control of a U6 promoter. For an all-in-one vector approach, the sgRNA sequence was set under a U6 promoter and the CD123 CAR was expressed under the Tet-On T11 promoter followed by iTom (Figure 4A). The Tet-On T11 promoter was used for consistency with previous work^4^ and was used in its “on” status for all experiments. For transduction of primary T cells and NFAT cell line, the CD123 CAR was cloned into a gamma-retroviral (gRV) vector under control of an RSV promoter, again in combination with iTom. A dTomato-only gRV construct was used as control vector. A functional human CD123 CAR (hCD123 CAR) was cloned in a lentiviral backbone under an SFFV promoter that was linked to a GFP reporter cassette. A *Bcl11b*-targeted shRNA was cloned in a lentiviral backbone under an SFFV promoter and linked to GFP.

Lentiviral supernatants were produced via transient transfection of 293T cells with the viral plasmids pMD2.G, pRSV.Rev, pcDNA.GP.4×CTE (plasmids produced by PlasmidFactory) and the respective transfer vector plasmids using the calcium phosphate transfection method. Harvested supernatant was filtered and concentrated via ultracentrifugation. The 58α-β-hybridoma cell line transduced with the M2 transactivator was used for viral titer determination of murine constructs.

Gamma-retroviral supernatant was produced by transient transfection of 293T cells with the viral plasmids pMD2.G (PlasmidFactory), pBS-CMV-gagpol (Addgene), and the respective CAR plasmid using the calcium phosphate transfection method. Primary T cells and the NFAT cell line were transduced with fresh viral supernatant.

### Generation of engineered murine lymphoid progenitors

Murine HSPCs (lin^−^) were isolated from BM with antibodies against lineage markers and sorted for c-kit^+^ and Sca-1^+^ (LSK) cells. LSKs were then transduced with concentrated lentiviral supernatant (MOI 50) and cultured as published.^67^ Briefly, transduced LSK cells were transferred to OP9-DL1 monolayer cells in complete αMEM medium (Life Technologies) supplemented with 10% heat-inactivated FCS, FLT3-L (5 ng/mL), IL-7 (5 ng/mL) (Peprotech), and Dox (2 mg/mL) (MilliporeSigma). Lymphoid progenitors were transferred to new OP9-DL1 monolayers every 3 to 4 days. Transduced lymphoid progenitors were sorted on day 10–13 of OP9-DL1 co-culture for dTomato expression (Tom^+^) and frozen on days 13–20. For *in vivo* mouse studies, engineered lymphoid progenitors were thawed and cultured for a further 7 days on OP9-DL1 with cytokines. Dox was added during the entire culture period from day 1 onward. Engineered lymphoid progenitors between days 20 and 27 of co-culture were used for co-transplantation. Cultures were supplemented with Dox for permanent transgene expression unless otherwise noted.

### Primary human UCB samples and generation of human-engineered lymphoid progenitors

UCB samples were processed as described earlier.^3^ Briefly, purified CD34^+^ HSPCs were transduced with lentiviral supernatant and transferred onto OP9-DL1 stromal cells in the presence of hIL7 (10 ng/mL), hSCF (20 ng/mL), hTPO (20 ng/mL), and hFLT3L (10 ng/mL). Human-engineered lymphoid progenitors were harvested every 3–4 days and cultured onto new OP9-DL1 monolayers supplemented with respective cytokines.

### Flow cytometry and cell sorting

Single-cell suspensions of murine origin were stained with the following fluorochrome-conjugated antibodies: CD3 (Brilliant Violet [BV] 421; catalog 100336; clone 145-2c11), CD4 (BV 421; catalog 100437; clone GK1.5), CD8 (APC; catalog 100712; clone 53-6.7), TCR-β (PE-Cy7; catalog 109222; clone H57-597), CD25 (BV421/PeCy7; catalog 102033/102016; clone PC61), CD44 (APC; catalog 103012; clone IM7), NK1.1 (APC; catalog 108710; clone PK136), NKp46 (BV 421; catalog 137612; clone 29A1.4), IFN-γ (APC; catalog 505809; clone XMG1.2), CD107a (APC; catalog 121613; clone 1D4B), NOTCH1 (APC; catalog 130613; clone HMN-1-12), CD123 (Pe; catalog 106005; clone 5B11), Annexin (APC; catalog 640941), CD45 (APC; catalog 109814; clon3 104), Gr.1 (FITC; catalog 108406; clone RB6-8C5), CD11b (PeCy7; catalog 101215; clone M1/70), CD19 (PeCy7; catalog 115520; clone 6D5), CCR9 (PeCy7; catalog 128711; CW-1.2), CD49a (PeCy7, catalog 108926; clone Dx5, γδTCR (PeCy7; catalog 118124; clone GL3) (all from BioLegend), CD123 (APC; catalog 4330936; clone 5B11) (from Invitrogen), and PI (catalog 51-66211E) (from BD Bioscience). For binding assays of phage display-derived mCD123 scFv, His-labeled scFvs were stained with anti-His antibodies (Pe; catalog 130-0899-810) (from Milteny Biotec). For lineage^–^c-kit^+^Sca-1^+^ sort, antibodies against lineage markers CD3, CD4, CD8, CD19, NK1.1, Gr-1, and CD11b in FITC (catalog 100306/100406/100706/115506/108706/108406/101206; clone 145-2CM/GK1-5/53-6.7/6D5/DM136/RB6-8C5/M1/70) or Pe (catalog 100308/100408/100708/115508/108708/108408/101208) and Sca-1 (Pe/BV-421; catalog 108108/108128; clone D7) (all from BioLegend) and c-kit (APC; catalog 17-1172-83; clone ACK2) (eBioscience) were used. For CAR expression detection, cells were stained with protein L (GenScript; catalog M00097) and detected by APC-labeled streptavidin (BD Biosciences; catalog 554067). Human cells were stained with the following antibodies after blocking with Human TruStain FcX (catalog 422302): CD34 (PE-Cy7; catalog 343516; clone 581), CD5 (BV421; catalog 300626, clone UCHT2), CD1a (APC; catalog 300110; clone HI49), NOTCH1 (APC; catalog 352107; clone MHN1-519), CD7 (PeCy7; catalog 343113; clone CD7-6B7), CD161 (BV421; catalog 339913; clone HP-3G10), NKp46 (APC; catalog 331917; clone 9E2), CD16 (PeCy7; catalog 360707; clone B73.1), NKG2D (BV421; catalog 320821; clone 1D11), and CD56 (BV421; catalog 318328; clone HCD56) from BioLegend and CD7 (APC; catalog 561604; clone M-T701) from BD Bioscience. For human CD123 CAR staining, human CD123 peptide with a His tag (Sino Biologicals, Beijing, China) and an anti-His tag antibody (APC; catalog IC050A) from R6D Systems were used. Flow cytometry was performed using a FACSCanto or LSR-II (BD Biosciences), and the data were analyzed with FlowJo software (BD). Flow cytometry analysis was based on fluorescence minus one (FMO) controls. Relative numbers from thymi, BM, and splenocytes were calculated from Tom^+^ gate. Relative cell numbers from PB were calculated by leukocyte cell numbers detected by blood cell counter (ScilVet ABC Animal Blood Counter) and frequencies of lineage marker from flow cytometry (CD19^+^ = B cells, Gr.1^+^CD11b^+^ = granulocytes, CD3^+^ = T cells).

### Intracellular cytokine staining and degranulation assay

Single-cell suspensions of donor B6 splenocytes were harvested and T cells were expanded using CD3/CD28 (BioLegend) antibodies in the presence of recombinant IL-2 (20 U/mL) and IL-7 (4 ng/mL). Primary murine T cells were cultured in RPMI 1640 medium supplemented with 10% heat-inactivated FCS (Capricorn), l-glutamine, HEPES buffer, and penicillin/streptomycin (all Gibco, Thermo Fisher Scientific). T cells were transduced twice on RetroNectin-coated (Takara) plates with fresh gamma-retroviral supernatant. After 4 days, T cells were co-incubated with C1498-mCD123 cells and intracellular IFN-γ staining was performed. For detection of degranulation, T cells were co-incubated on 96-well plates with C1498-mCD123 cells at a 10:1 ratio in the presence of an anti-CD107a antibody. After 1 h, GolgiStop (BD Bioscience) was added. Cells were harvested after 4 h and treated with the CytoFix/CytoPerm Kit (BD Bioscience) for flow cytometry analysis.

### NFAT signaling assay

The NFAT cell line expressing an NFAT-inducible GFP reporter construct was used to measure CAR signaling activity by GFP detection. The CD123 CAR construct and iTom control were brought into the NFAT cells by γRV transduction. The cells were stimulated by co-culturing with irradiated C1498/-mCD123 cells in a 10:1 ratio (0.6 × 10^6^ NFAT, 0.6 × 10^5^ C1498/-mCD123). After 24-h incubation, GFP expression was detected by flow cytometry.

### Cytotoxicity assay

Cytotoxicity assays on target C1498 or C1498-mCD123 cells were performed using the JAM assay.^68^ Briefly, [3H]thymidine (Amersham, Woburn, MA) labeled C1498 or C1498-mCD123 cells were co-incubated with effector cells (im12328z1 primary T cells or lymphoid progenitors) in a serial dilution of effector-target ratios starting at 50:1. After 4 h of co-incubation, the cells were harvested and the residual cellular radioactivity determined. Percentage specific killing of target cells was calculated using the following formula: % specific killing = (S − E/S) × 100, where E is the radioactivity in the presence of effector (in cpm) and S the radioactivity in the absence of effectors (spontaneous). Data are presented as the mean percentage specific killing of triplicate samples (±SE) from representative experiments.

To evaluate cytotoxic activity by FACS, C1498 or C1498-mCD123 target cells were exposed to effector lymphoid progenitor cells for 24 h, followed by flow cytometric analysis of target cell viability. In brief, target cells were labeled with CellTrace Violet Cell Proliferation Dye according to the manufacturer’s instructions (Invitrogen, Waltham, MS USA) and seeded into 96-well plates at a density of 50,000 cells/well. Next, effector T cells were added at different E:T ratios (0.5:1, 1:1, 5:1, 10:1, 20:1). Target cells cultured in absence of effector T cells served as control for baseline target cell viability. After 24 h, the cells were harvested and washed with PBS, followed by determination of target cell viability via flow cytometry using 7-aminoactinomycin D (7-AAD, BD Biosciences, Heidelberg, Germany) staining. Samples were acquired using the BD FACSCanto 10c system (BD Biosciences, Heidelberg, Germany). Flow cytometry data were analyzed using FlowJo v10 (FlowJo LLC, BD Biosciences) and visualized using Prism Version 10 (GraphPad Software, San Diego, USA).

### Cytokine, chemokine, and growth factor detection in culture supernatants

Engineered murine lymphoid progenitors were stimulated on day 20 of *in vitro* differentiation for 24 h with either C1498 or C1498_mCD123 target cells or remained unstimulated. Cytokine, chemokine, and growth factor concentrations in supernatants were quantified by the Luminex-based multiplex technique according to the manufacturer’s instructions (Bio-Rad, USA). The mouse 23-plex (M60009RDPD, Bio-Rad, Hercules USA) was used with 50 μL of culture supernatant. Standard curves and concentrations were calculated with Bio-Plex Manager 6.2, and the detection sensitivity of all proteins was between 1 pg/mL and 10 μg/mL.

### BM and lymphoid progenitor co-transplantation and leukemia challenge

Total body irradiation of 8-week-old female B6 recipients was performed with 10.5 Gy from a linear accelerator. After 24 h, mice were co-transplanted with 3 × 10^6^ TCD-BM and 8 × 10^6^ lymphoid progenitors as previously described.^67^ In order to set the CAR-transgene expression in the “on” status from the very beginning of the experiment, all *in vivo* studies were performed under permanent administration of Dox-containing water or food for transgene expression. For leukemia studies, 1 × 10^6^ C1498-mCD123-GFP leukemia cells were injected via the lateral tail vein on day 21 after transplantation. All mice were randomly assigned to experimental groups, and no blinding of investigators was performed.

### *Ex vivo* short-term culture

Splenocytes from co-transplanted mice were harvested 12 days after transplantation and brought into short-term culture under T cell-supporting conditions with ConA (5 mg/mL) (MilliporeSigma), IL-7 (5 ng/mL), and IL-2 (20 U/mL) (Peprotech) or cultured under CARiK cell-supporting conditions with high IL-2 concentrations (1,000 U/mL). Cells were passaged every 2 days and were assessed for Tom^+^ frequencies after 4 days of culture.

### Western blotting

Cell lysates of transgene-positive sorted lymphoid progenitors or WT B6 thymocytes were prepared in RIPA buffer. Equal amounts of protein lysates were separated by SDS-PAGE and transferred onto polyvinylidene fluoride (PVDF) membranes (Amersham). Membranes were blocked with 5% skim milk, and BCL11B was stained with rat anti-mouse primary antibody (clone 25B6; BioLegend) and detected with HRP-coupled goat anti-rat secondary antibody (Poly4054; BioLegend).

### PCR for rearrangement on the TCR-β locus

Murine engineered lymphoid progenitors were harvested from *in vitro* culture and sorted for Tom^+^ cells. Genomic DNA was isolated (Qiagen) and D-J rearrangement at the *Tcrb* locus assessed via PCR using TCRB_Jβ2, reverse, TGAGAGCTGTCTCCTACTATCGATT and TCRB_Dβ2, forward, GTAGGCACCTGTGGGGAAGAAACT as primers (5′–3′) as described^11^ for mouse TCR rearrangement.

### RNA extraction and reverse-transcription qPCR

On day 20 (murine) or day 14 (human) of OP9-DL1 co-culture, RNA from sorted samples was extracted using the RNAeasy Mini Kit (QIAGEN) and converted into cDNA using the QuantiTect Reverse Transcription Kit (QIAGEN). Real-time PCR reactions were performed using the QuantiTec SYBR Green PCR kit (QIAGEN) on an Applied Biosystems 7300 Real-Time PCR System (Thermo Fisher Scientific). The QuantiTec Primer assays for BCL11B (murine: QT00285663, human: QT00080983) and ACTB (murine, QT249900; human, QT00095431) were purchased from QIAGEN. Relative expression of BCL11B was normalized to ACTB. Non-transduced human CD34^+^ cells and murine iTom-transduced lymphoid progenitors were used for controls.

### Microscopy

Thymi of mice co-transplanted with engineered lymphoid progenitors were harvested on day 12 after transplantation. Sections from Tissue-Tec OCT (Sakura)-embedded thymi were analyzed with a Zeiss Axio Imager 2 microscope (×20 magnification) and acquired with ZEN pro software (Zeiss). Images were equally processed with ZEN lite software.

### Vector copy number determination by ddPCR

For mVCN determination, genomic DNA (gDNA) was isolated (Qiagen). VCN was determined on 50 ng of isolated gDNA with a Taqman approach on a QX200 ddPCR system (Bio-Rad, Feldkirchen, Germany) as previously described.^69^ The number of viral sequences was normalized to the genomic reference sequence of polypyrimidine tract binding protein 2 (Ptbp2). Primer pairs and probes for the wPRE element (viral vector detection; wPRE-forward, 5′-GAGGAGTTGTGGCCCGTTGT-3’; wPRE-reverse, 5′- TGACAGGTGGTGGCAATGCC-3’; wPRE-probe, 5′-FAM-CTGTGTTTGCTGACGCAAC-BHQ1-3′) and for Ptbp2 (PTBP2-forward, 5′-TCTCCATTCCCTATGTTCATGC-3’; PTBP2-reverse, 5′- GTTCCCGCAGAATGGTGAGGTG-3’; PTBP2-probe, 5′-JOE- ATGTTCCTCGGACCAACTTG-BHQ1-3′) were used in a 20-μL qPCR reaction using the ddPCR system and following the manufacturer’s instructions. The amplified products were measured with the QX200 droplet reader, and the concentration of each target per microliter and resulting VCN values were determined with the QuantaSoft software (Bio-Rad). The threshold for droplet positivity was manually adjusted in each experiment following the manufacturer’s recommendations, as it was not automatically recognized in some cases. For the *ex vivo* samples from batch 2, a qPCR approach using the StepOnePlus thermocycler (Applied Biosystems, Carlsbad, CA, USA) was performed, also Taqman based using the same primer/probe pairs as for ddPCR and following the manufacturer’s instructions. Reactions were prepared in MicroAmp Fast Optical 96-Well Reaction Plates and sealed with MicroAmp Optical Adhesive Film (Applied Biosystems). Serial dilutions of a linearized plasmid standard containing both targets (wPRE and PTBP2) were used to calculate a standard curve for the final VCN determination.

### ISA with INSPIIRED

Vector insertion sites were determined using the INSPIIRED (Integration Site Pipeline for paired-end reads) pipeline as described before.^36^^,^^70^ Samples with very low gDNA amounts after isolation were first amplified with the whole-genome amplification (WGA) REPLI-g mini kit (Qiagen) following the manufacturer’s instructions. For INSPIIRED, between 70 and 1,000 ng of native or WGA-amplified gDNA were sonicated using the S220 Focused-ultrasonicator (Covaris, Woburn, MA, USA), purified using AMPure beads in a 0.7-fold ratio, and end-repaired with the NEBNext Ultra II End Repair/dA-Tailing module (New England Biolabs [NEB], Frankfurt, Germany). Previously generated specific linkers were ligated to the end-repaired DNA samples with the NEBNext Ultra II Ligation Module (NEB). After another round of purification using AMPure beads (0.7-fold bead-to-sample ratio), nested PCRs were performed to amplify the vector-genome junctions, adding Illumina adapter sequences and using specific index primers and sample-specific linker primers. The PCR products were visualized on 2% agarose gels, measured by Qubit, and pooled. The libraries were sequenced with an Illumina MiSeq sequencer using flow cells with 1 million clusters. Linker and primer sequences used for PCR reactions are available upon request.

Downstream bioinformatic processing was generally performed as described by Berry and colleagues.^36^ The analysis files necessary to run the INSPIIRED pipeline were downloaded from GitHub (https://github.com/BushmanLab/INSPIIRED). Demultiplexed sequences in FASTQ format were generated according to the individual index primers used, quality checked, aligned, and annotated to the mouse genome (mm9). The plasmid vector sequences served as a reference for long terminal repeat (LTR) regions and vector trimming. The processing and alignment statistics were exported before uploading the results to a local database. After creating a sample management database, reports with all integration site data were generated and used for customized post-processing in Excel and GraphPad Prism (GraphPad Prism, San Diego, CA, USA).

### Reverse transcription of RNA and quantification by ddPCR

RNA was isolated using the RNAeasy Mini Kit (Qiagen) and reverse transcribed with the QuantiTect Reverse Transcription Kit (Qiagen) following the manufacturer’s instructions. Probes and primer pairs targeting Mef2c (Mm.PT.58.9749652), Lmo2 (Mm.PT.58.31113581), and the housekeeping gene Actb (Mm.PT.39a.22214843.g) were purchased from Integrated DNA Technologies (IDT, Coralville, IA, USA) and used in a duplex qPCR reaction for quantification of the cDNA products using the ddPCR device (Bio-Rad) as described before.^71^ The concentration of each target per microliter was analyzed using the QuantaSoft software (Bio-Rad). The threshold for droplet positivity was manually adjusted in each experiment following the manufacturer’s recommendations. The concentrations of Mef2c and Lmo2 for each transduced sample were normalized by the housekeeping gene and plotted as absolute values in molecules/microliter.

### Microarray acquisition and processing

Microarrays using the isolated RNA were performed by the RCUG team of Hannover Medical School and as previously described in detail in Schwarzer et al.^37^ and Bastone et al.^38^ Briefly, when possible, 100 ng (or less, if not available) of total RNA were used to prepare Aminoallyl-UTP-modified (aaUTP) cRNA (Amino Allyl MessageAmp II Kit; Thermo Fisher Scientific), applying one round of amplification as directed by the company, except for a 2-fold downscaling of all reaction volumes. Before the reverse-transcription reaction, 1 μL of 1:5,000 dilution of Agilent’s One-Color Spike-In Kit stock solution (Agilent Technologies) was added to the total RNA used for each sample. The labeling of aaUTP-cRNA was performed with Alexa Fluor 555 Reactive Dye (Thermo Fisher Scientific) following the manufacturer’s instructions with the Amino Allyl MessageAmp II Kit (2-fold downscaled reaction volumes). Afterward, cRNA fragmentation, hybridization, and washing steps were carried out as recommended in One-Color Microarray-Based Gene Expression Analysis Protocol V5.7, except that 500 ng of each fluorescently labeled cRNA population was used for hybridization. Slides were scanned using the Agilent Micro Array Scanner G2565CA (pixel resolution 3 μm, bit depth 20). Data extraction was performed with the FeatureExtraction Software V10.7.3.1 using the extraction protocol file‘GE1_107_Sep09.xml.

### SAGA-XL analysis and SAGA-XL-GSEA

The potential genotoxicity due to insertional mutagenesis was investigated using the SAGA-XL pipeline described in detail in Bastone et al.^38^ Briefly, the samples were read in, quantile normalized, averaged, log_2_ transformed with the R package limma,^72^ and add-on batch corrected with the ComBat algorithm from the R package sva^73^ to previously generated lymphoid data consisting of untransduced controls, and samples were transduced with mutagenic (RSF91, SIN-LV.LMO2) or safer vector designs (SIN-LV.EFS). The genotoxicity predictors found in bulk cultures and in a subpopulation identified as the main driver of the immortalized phenotype (DN2 cells) were used to predict the new samples using a support vector machine with radial kernel by means of the R package caret.^74^ For visualization of the classification, PCAs were performed with the prcomp function from the R package stats, and the data were plotted in the first two principal components with the R package ggplot2.^75^ All calculations were performed in R 4.0.5 on a server running in Ubuntu 20.04.3. To check the enrichment of the oncogenic signature in the transduced samples, the SAGA-XL-GSEA approach was implemented, as described in Bastone et al.^38^ After running GSEA for the single samples against all MOCK, with B = 2,000 and Bonferroni-Holm correction from the phenoTest (Evarist Planet 2022) package, the samples with NES > 1.00 were considered as transforming.

### CAST-seq and ICE analyses

The CAST-seq libraries were prepared following the methods described in a previous study.^76^ PCRI and PCRII reactions were carried out using KAPA HiFi Hotstart Polymerase (Roche, #07958927001). Overall, three CAST-seq libraries from edited cells were prepared and compared to a single library generated from untreated control cells. NGS libraries were sequenced by GeneWiz (division of Azenta Life Sciences) using an Illumina NovaSeq 6000 device generating 2×150-bp paired-end reads. For the bioinformatics analysis, a modified version of the T-CAST pipeline,^77^ originally developed for analyzing TALEN-edited samples, was employed to analyze CRISPR-Cas-edited samples instead. Unspecific reads were filtered out based on their low read:hit ratio (<10). To assess CRISPR-Cas9 nuclease activity, the *Bcl11b* on-target site was amplified from genomic DNA of both edited and unedited (control) cells and the resulting PCR fragments subjected to Sanger sequencing. ICE software (https://doi.org/10.1089/crispr.2021.0113) was used to assess nuclease-induced indel formation.

### scRNA-seq

#### Library construction and sequencing

Splenocytes from co-transplanted mice were harvested 12 days post transplantation. Respective *ex vivo* progeny was labeled with Hashtag Antibodies (TotalSeq-B0301-4 anti-mouse Hashtag 1–4 Antibody, BioLegend; catalog numbers 155831, 155833, 155835, 155837). Cells were sorted for Tom+/7AAD− (BioLegend; catalog number 420404) using an Aria III fluorescence-activated cell sorter (BD). The single-cell suspension was then loaded onto a well on a 10X Chromium Controller (10× Genomics), targeting 10,000 cells for encapsulation. cDNA libraries were constructed using the 10× Chromium Single Cell 3′ Library Kit following the manufacturer’s protocol. Libraries were sequenced on an Illumina NovaSeq 6000 with 2 × 50 paired-end kits, using the following read lengths: 16 bp for read 1 (R1) containing the 10× barcode and unique molecular identifier (UMI), 8 bp for the sample index, and 89 bp for read 2 (R2) covering the transcript. Data were processed using Cellranger 7.01: first, sequenced samples were demultiplexed with “cellranger mkfastq,” followed by alignment of read 2 to the mouse reference genome (mm10) using STAR via “cellranger count.” Only reads with valid barcodes and UMIs that mapped to exons and introns (Ensembl GTFs GRCm38.p4) and did not contain PCR duplicates were included. Valid cell barcodes were determined based on UMI distribution.

#### Sample demultiplexing and data integration

Single-cell data analysis was performed using R Studio 2022.02.02 and Seurat v5.0.33,4 with R version 4.2.1 5. Hash-tagged samples were demultiplexed using the HTODemux() function on k-medoid clustered normalized HTO values, with a 0.95 quantile threshold used for sample classification. Cells positive for more than one HTO were labeled as doublets and excluded from the analysis. Additionally, cells with low feature counts (nFeature < 200) or high mitochondrial content (>20%) were filtered out. Pre-processed data were loaded sample-wise into individual Seurat objects and normalized using the SCTransform() function. Data integration was performed using SelectIntegrationFeatures(), PrepSCTIntegration(), FindIntegrationAnchors(), and IntegrateData() functions with default parameters. Dimensionality reduction was conducted on the integrated dataset using RunPCA(dims = 1:30).

#### Cell type annotation via reference-based label transfer/mapping

A well-established CITE-seq dataset of human PBMCs^78^ was utilized to transfer cell type annotations to murine splenocytes. For this cross-species analysis, the human reference and murine query datasets were refined to include only 1:1 orthologous genes. The human reference dataset was re-normalized and integrated following the methods described by Hao et al.^78^ Anchors between the human reference and murine query samples were identified using the FindTransferAnchors() function. Mapping scores for each cell were calculated using the MappingScore() function, providing a confidence value between 0 and 1, indicating the degree of representation of a murine cell by the human reference dataset. Cell-type labels were transferred, and the query data were projected onto the UMAP structure of the reference using the MapQuery() function.

#### Unsupervised clustering

To identify clusters with similar expression profiles, the FindNeighbors() and FindClusters(resolution = 0.1) functions were applied. Marker genes for each cluster were identified using the FindAllMarkers(only.pos = T) function.

#### Differential gene expression analysis

Each sample was compared to the control sample (iTom) using the FindMarkers() function, which compared normalized gene expression values (SCT normalized). Differentially expressed genes were identified by an absolute log2 fold change >1 and a false discovery rate (FDR) <0.05, based on the Wilcoxon signed-rank test. Average gene expression values per sample were calculated using the AverageExpression() function. GSEA was conducted using the R package clusterProfiler (v2.1.6)6.

### Statistics

Statistics were performed and graphed with GraphPad Prism 5 software for Mac (GraphPad Software). Survival curves were compared using Mantel-Cox (log rank) test. Student’s t test (two-tailed) was applied for two-group comparisons and one-way ANOVA with Tukey’s *post hoc* test for comparing more than two groups. Data were represented as mean ± SEM. *p* values of less than 0.05 were considered to be significant.

### Study approval

Human UCB was used after local ethic committee approval and consent of the child’s parents. All animal experiments were approved by the State Government of Lower Saxony, Germany (approval code 33.19-42502-04-20/3446) and performed in accordance with institutional animal care and use guidelines.

## Data availability

All original microarray data were deposited in the NCBI’s Gene Expression Omnibus database (GEO GSE252499) https://www.ncbi.nlm.nih.gov/geo/query/acc.cgi?acc=GSE252499. Enter token srwhswuqzlczheb into the box.

## Acknowledgments

We thank the staff of the Cell Sorting Core Facility (Hannover Medical School) and the members of the Genome Analytics Facility of the Helmholtz Centre for Infection Research (Braunschweig) for their assistance. Many thanks to Michael Morgan who edited the final version of the manuscript as a native speaker. This work was supported by 10.13039/501100001659Deutsche Forschungsgemeinschaft (SA 1371/6-1 to M.G.S. and HU 1715/8-1 to M.H.), and the PhD program Molecular Medicine of the 10.13039/501100005624Hannover Medical School (HBRS).

## Author contributions

F.B. designed research, performed experiments, analyzed and interpreted data, and drafted and edited the manuscript. J.H., A.L.B., S.P. and M. Rhiel performed experiments. J.M., J.F., T.M., M.H., R.G., M. Rothe, A.L.B., M. Rhiel, B.E.-V., and T.C. contributed vital new technology and reagents. A.G. and A.S. analyzed and interpreted data. A.G., A.S., T.M., M. Rhiel, T.C., and M.R.M.v.d.B. edited the manuscript. M.G.S. designed the general concept and research, analyzed and interpreted data, and drafted and edited the manuscript.

## Declaration of interests

The authors declare no competing interests.

## Supplemental information

Document S1. Figures S1–S8 and Tables S1 and S2

Document S2. Article plus supplemental information
