# Supplementary material for: Targeting BCL11B in CAR-engineered lymphoid progenitors drives NK-like cell development with prolonged anti-leukemic activity
Source: Mol Ther. 2025 Feb 15;33(4):1584–607. doi: 10.1016/j.ymthe.2025.02.024 (PMC11997514; doi:10.1016/j.ymthe.2025.02.024)
Supplement: Document S1. Figures S1–S8 and Tables S1 and S2 [file mmc1.pdf]

YMTHE, Volume *33*

Supplemental Information

Targeting BCL11B in CAR-engineered lymphoid progenitors drives NK-like cell development with prolonged anti-leukemic activity

Franziska Baatz, Arnab Ghosh, Jessica Herbst, Saskia Polten, Johann Meyer, Manuel Rhiel, Tobias Maetzig, Robert Geffers, Michael Rothe, Antonella Lucia Bastone, Philipp John-Neek, Jörg Frühauf, Britta Eiz-Vesper, Agnes Bonifacius, Christine S. Falk, Constantin v. Kaisenberg, Toni Cathomen, Axel Schambach, Marcel R.M. van den Brink, Michael Hust, and Martin G. Sauer

Supplemental Information

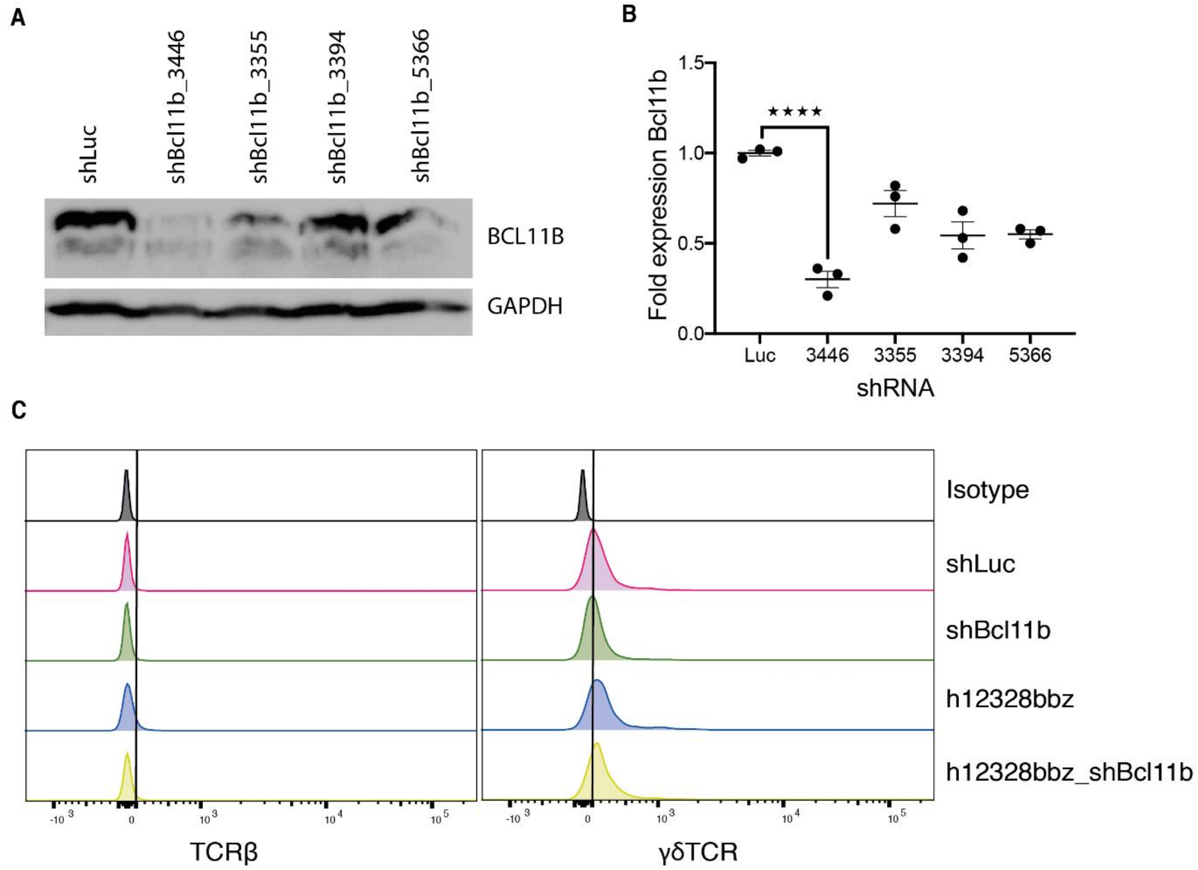

Figure S1: Degree of *Bcl11b* knock down by four different *Bcl11b*-targeting shRNAs and impact of BCL11B suppression on T cell development. (**A, B**) Four *Bcl11b*-targeted shRNA sequences were comparatively assessed for their gene knockdown efficiency. An irrelevant Luciferase-targeted shRNA served a control. (**A**) Western blot analysis of BCL11B in lysates from shRNA-transduced Jurkat cells. (**B**) qPCR analysis of *Bcl11b* expression in shRNA-transduced Jurkat cells. Respective results from 1 of 2 independent experiments are shown. (**C**) Human CD34$^+$ UCB-derived HSPCs were engineered with respective constructs and consecutively differentiated on OP9-DL1 stromal cells. Flow cytometry analysis was performed on day 14 of culture gating GFP$^+$ cells. Respective results from 1 of 2 independent experiments are shown. Histograms represent TCRβ and γδTCR expression on engineered lymphoid progenitors. Statistics was performed using 1-way ANOVA with

Tukey´s post test. Each data point represents an individual sample. Data are shown as mean ±SEM. *P<0.05; ** P<0.01; ***P<0.001; ****P<0.0001.

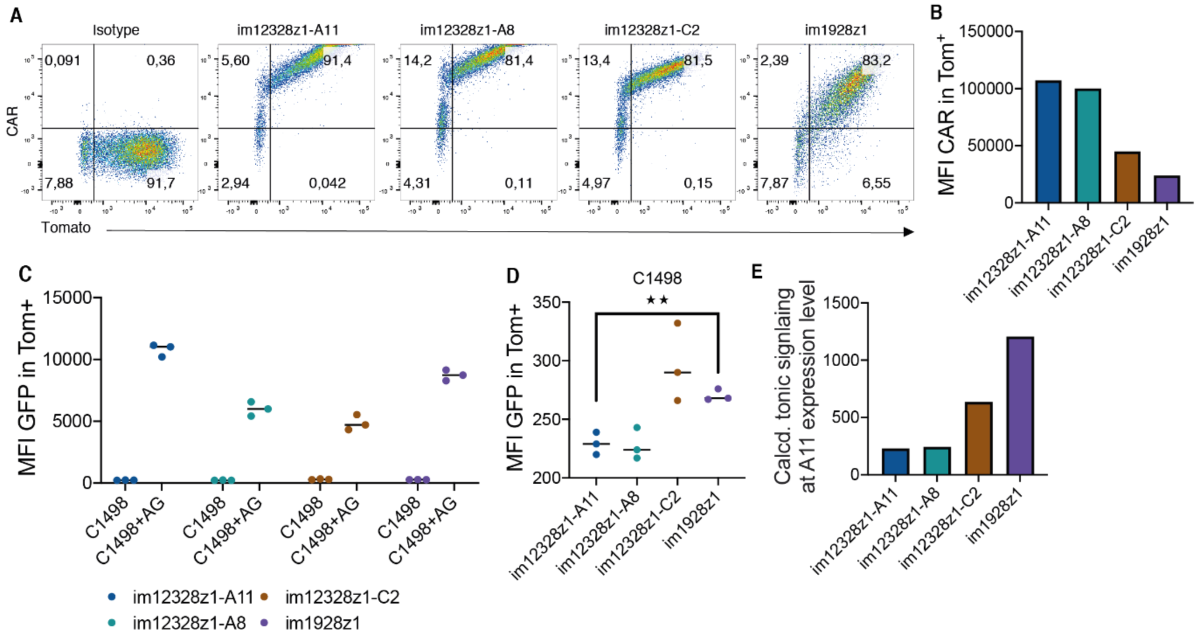

Figure S2: CD123 CARs have lower tonic signaling activity than a previously published murine CD19 CAR. (**A, B**) CAR expression was assessed by flow cytometry on transduced NFAT cells using protein L staining. (**C**) Intracellular signal strength (GFP expression) of CAR-transduced NFAT cells upon stimulation with either C1498-mCD123/mCD19 or C1498 control cells was assessed by flow cytometry. (**D**) Tonic signaling activity after stimulation with antigen-negative C1498 cells. (**E**) Extrapolating calculation of tonic signaling normalized for the CAR expression level of A11. Respective results from 1 of 2 independent experiments are shown. Each data point represents an individual sample. 1-way ANOVA with Tukey´s post test was used for analysis. *P<0.05; ** P<0.01; ***P<0.001; ****P<0.0001.

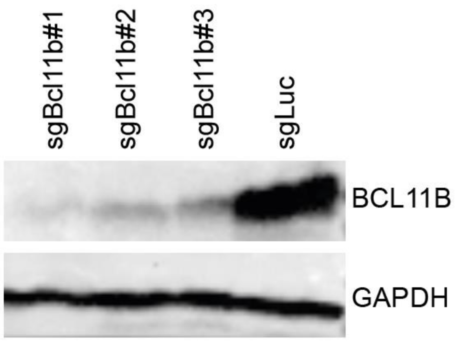

Figure S3: Comparative analysis of three different *Bcl11b*-targeting sgRNAs. Western blot analysis of BCL11B in lysates of transduced lymphoid progenitor cells. Three *Bcl11b*-targeted sgRNA sequences were comparatively assessed for their gene knockout efficiency. An unrelated Luciferase-targeted sgRNA served a control. The experiment has been performed once.

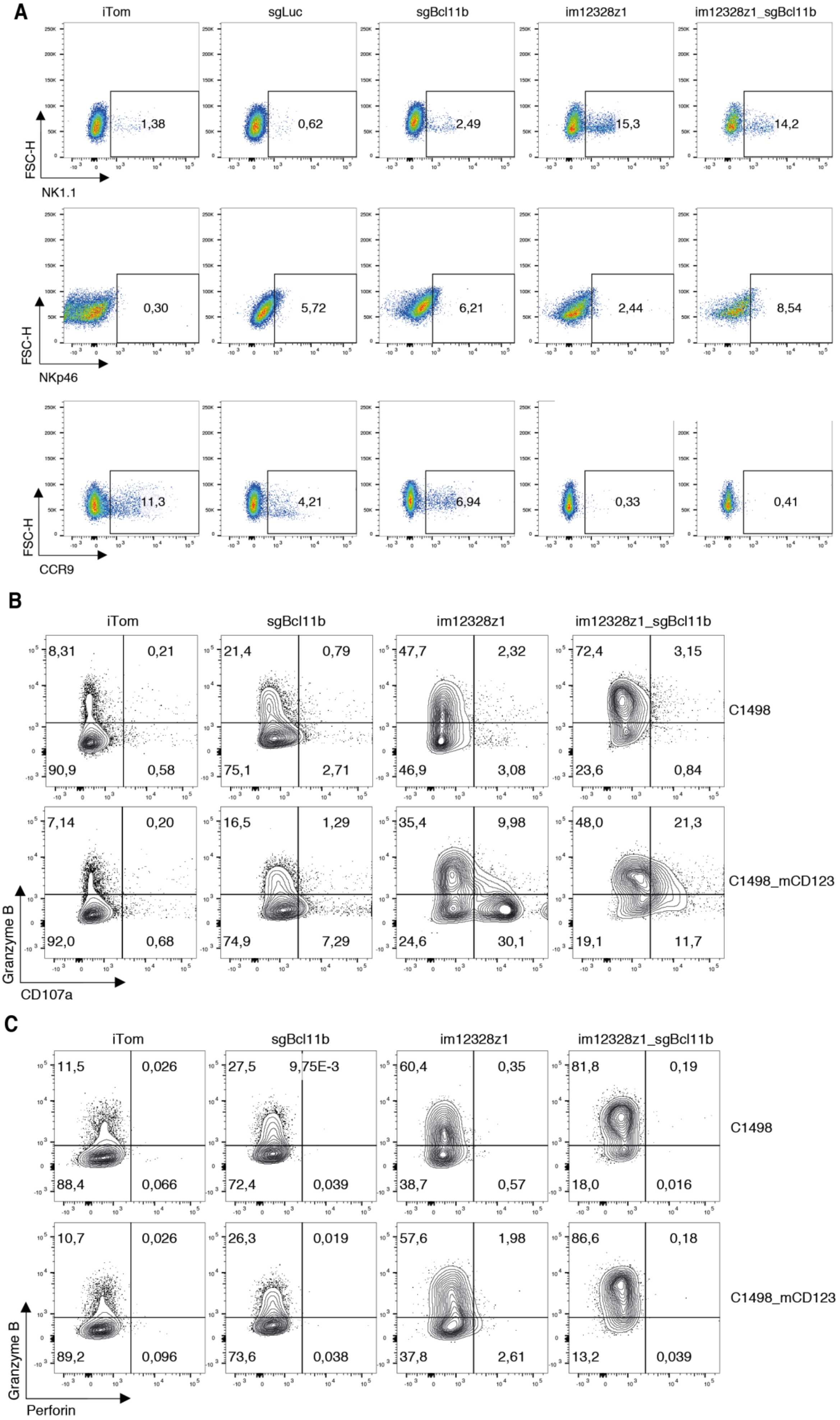
A
iTom
sgLuc
sgBcl11b
im12328z1
im12328z1_sgBcl11b
FSC-H
NK1.1
1,38
0,62
2,49
15,3
14,2
NKp46
0,30
5,72
6,21
2,44
8,54
CCR9
11,3
4,21
6,94
0,33
0,41
B
iTom
sgBcl11b
im12328z1
im12328z1_sgBcl11b
8,31 0,21 90,9 0,58
21,4 0,79 75,1 2,71
47,7 2,32 46,9 3,08
72,4 3,15 23,6 0,84
C1498
7,14 0,20 92,0 0,68
16,5 1,29 74,9 7,29
35,4 9,98 24,6 30,1
48,0 21,3 19,1 11,7
C1498_mCD123
Granzyme B
CD107a
C
iTom
sgBcl11b
im12328z1
im12328z1_sgBcl11b
11,5 0,026 88,4 0,066
27,5 9,75E-3 72,4 0,039
60,4 0,35 38,7 0,57
81,8 0,19 18,0 0,016
C1498
10,7 0,026 89,2 0,096
26,3 0,019 73,6 0,038
57,6 1,98 37,8 2,61
86,6 0,18 13,2 0,039
C1498_mCD123
Granzyme B
Perforin

Figure S4: NK cell marker, chemokine receptor and activation marker expression of engineered murine lymphoid progenitors. Murine LSK cells were transduced with lentiviral vectors and consecutively differentiated on OP9-DL1 stromal cells. Flow cytometry analysis was performed within the Tom^+ gate on day 20 of co-culture. (**A**) Representative flow cytometry plots of NK1.1, NKp46, and CCR9 expression. Specific responses of engineered lymphoid progenitors upon stimulation with C1498 or C1498_mCD123 target cells *in vitro* were measured by (**B**) CD107a degranulation, (**B, C**) granzyme B and (**C**) perforin. Representative results from 1 or 2 independent experiments are shown.

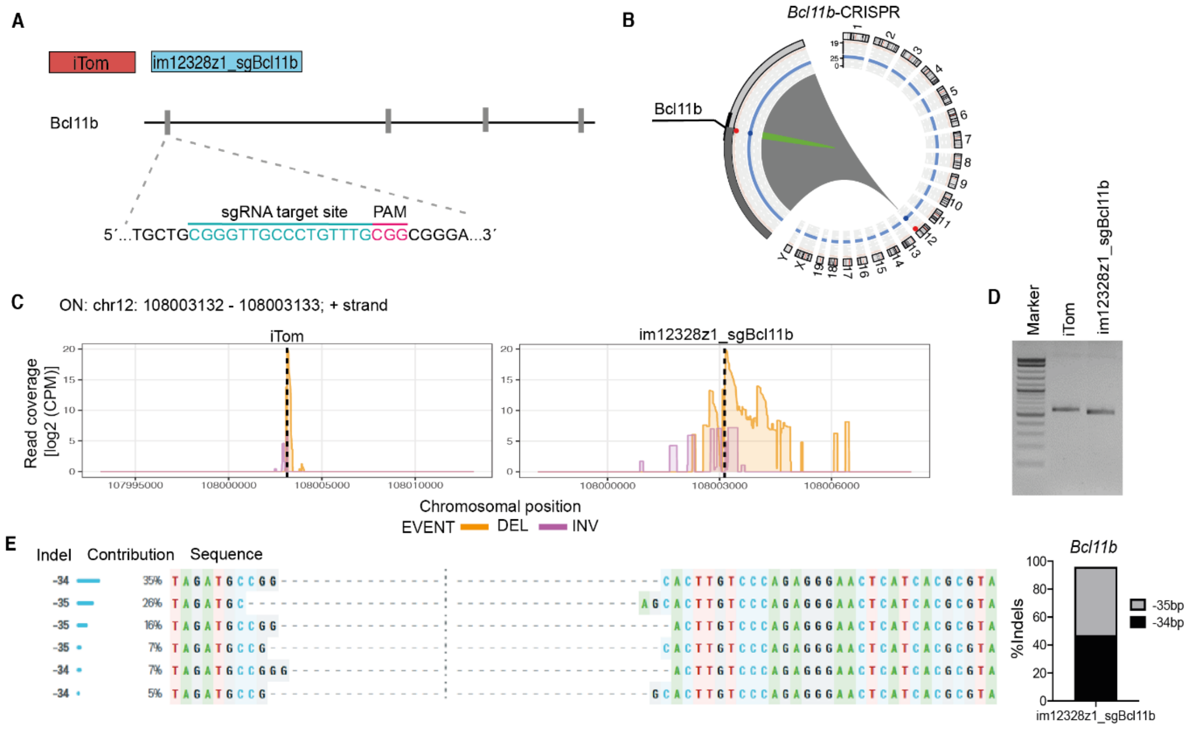

Figure S5: CAST-Seq reveals a favorable safety profile for *Bcl11b* knockout. Lymphoid progenitor cells were transduced with im12328z1_sgBcl11b or the iTom control vector**.** (**A**) Map of the CRISPR/Cas9 target site within the *Bcl11b* gene. (**B**) Structural variations. Circos plots illustrate CAST-Seq results of cells that had been transduced with im12328z1_sgBcl11b. The chromosome 12 region comprising the *Bcl11b* locus is enlarged and on-target site aberrations marked in green. (**C**) Coverage plot of the *Bcl11b* target site. Shown are the chromosomal position vs. the normalized number of reads indicating deletions (orange) and inversion (red), as well as the sgRNA binding sites (black dashed line). (**D**) Qualitative genotyping. The *Bcl11b* target site was amplified by PCR. (**E**) Quantitative genotyping. ICE analysis of Sanger sequencing data shows the fraction and the sequence of the various modified alleles. Experiment has been performed once.

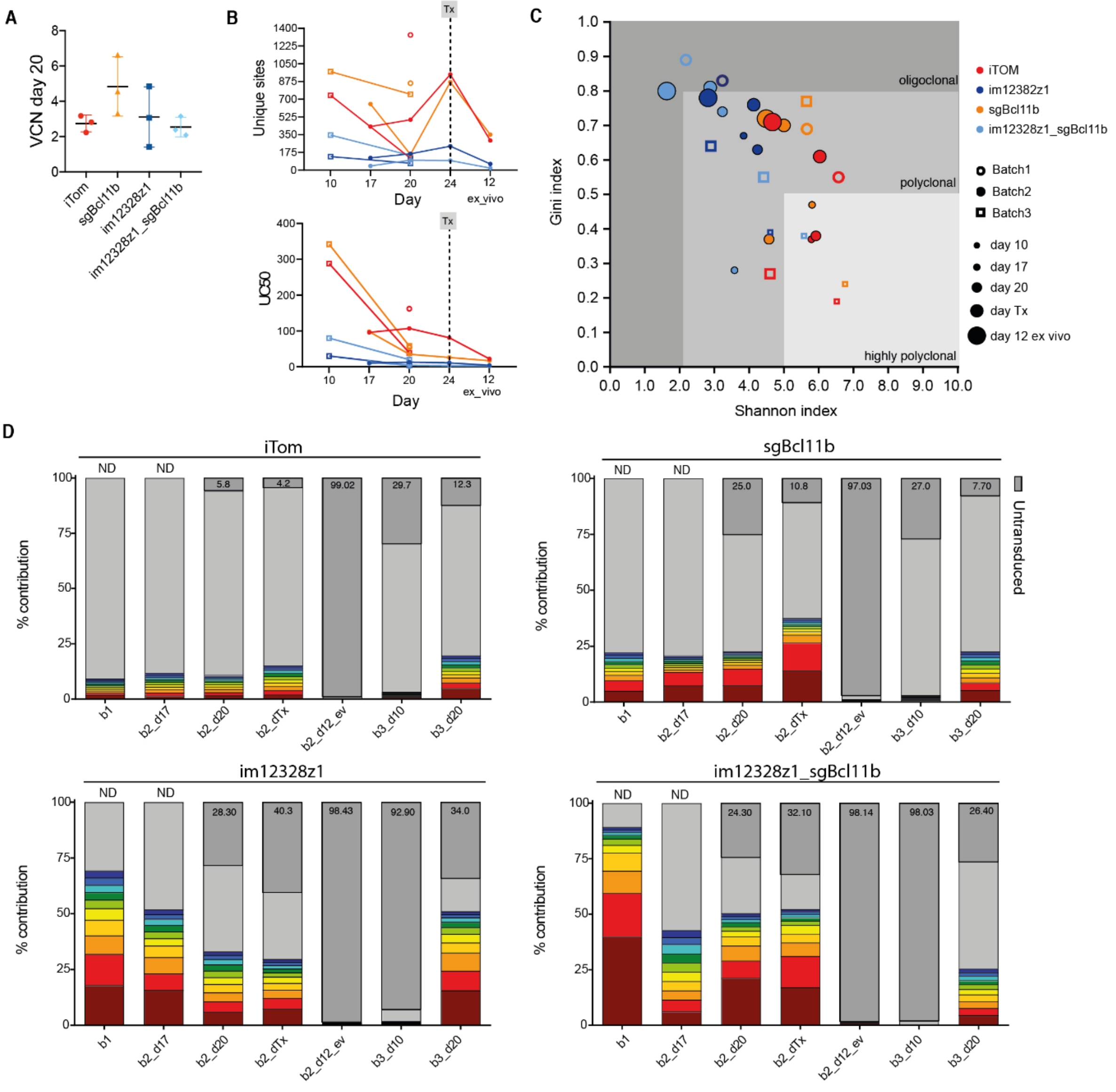

Figure S6: Insertion site analysis and clonal dynamics of gene-edited HSPCs over time. Murine LSK cells were transduced with sgBcl11b, im12328z1, im12328z1_sgBcl11b or the iTom control vector and differentiated into lymphoid progenitors using the OP9-DL1 co-culture system. ISA was performed during *in vitro* differentiation on day 17, 20 and immediately before co-transplantation on day 24 (3 independent *in vitro* cultures). Irradiated female B6 recipients were reconstituted with 3 x 10^6 B6 TCD-BM and co-transplantated with 8 x 10^6 engineered lymphoid progenitors (n=3-5 mice/group). Progeny cells were retrieved from the recipient and assessed for clonal evolution 12 days after adoptive transfer. ISA, insertion site analysis. (**A**) VCN determination by ddPCR of transduced lymphoid progenitor cells on

day 20 of *in vitro* differentiation. VCN, vector copy number; ddPCR, droplet digital PCR. (**B**) Number of unique insertion sites at indicated time points obtained after the alignment to the mouse genome and UC_{50} values of individual samples representing the amount of insertions covering 50% of the sequence pool. A low value suggests few integrations are overall dominant. (**C**) Cell clonality assessed by the Gini and the Shannon indices. Gini coefficients estimate the sequence abundance contribution, with high values indicating few insertions dominate the sequence pool. Shannon diversity indices reflect the diversity of the sequence pool. Low values suggest the sequence pool consists of few integrations. (**D**) Insertion site contribution normalized by the number of transduced cells. Each data point represents an individual sample. Data are shown as mean ±SEM.

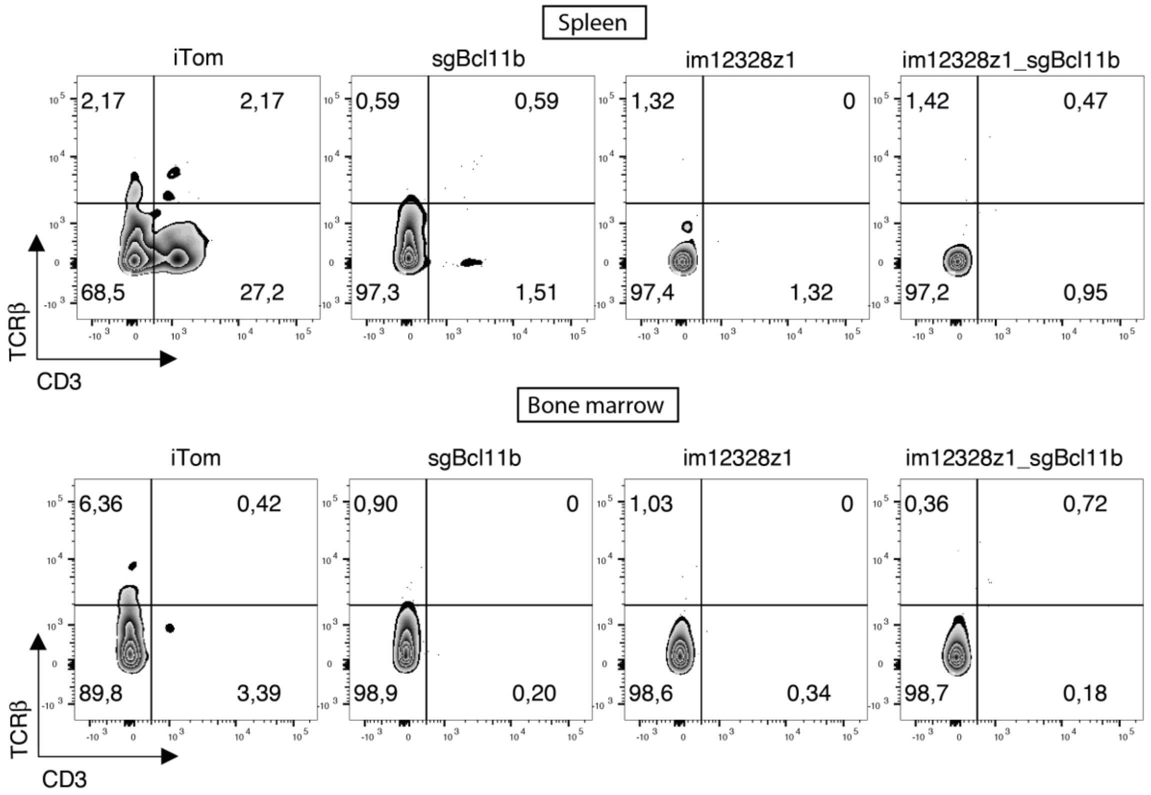

Figure S7: Comparative analysis of T cell development of engineered lymphoid progenitors after *in vivo* differentiation. Irradiated female B6 recipients were reconstituted with 3 x 10^6 B6 TCD-BM and con-transplanted with 8 x 10^6 engineered lymphoid progenitors (n=4 mice/group). 12 days after adoptive transfer, cells were analyzed in spleen and bone by flow cytometry after gating on Tom^+ cells. TCRβ and CD3 expression is shown. Experiment was performed once.

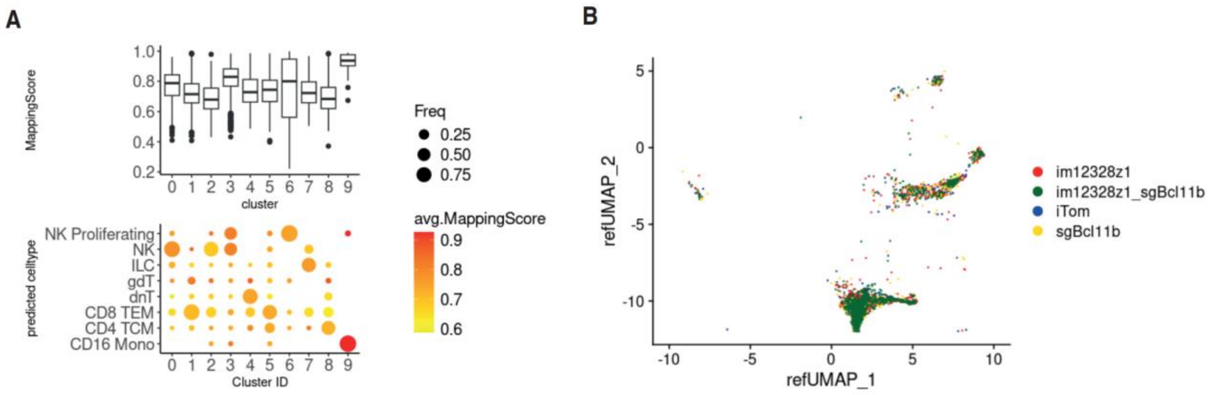

Figure S8: Reference-based cell type mapping using a CITE-seq dataset of human PBMCs. (**A**) The upper panel shows the distribution of Mapping Scores, which indicate the confidence level (0 to 1) of how well a murine cell is represented by the human reference dataset. The lower panel illustrates the enrichment of predicted cell types (circle size) and their corresponding average MappingScore (circle color) for each murine cell cluster. (**B**) Dimensional reduction plot displaying cell clusters identified from splenocytes with color code for each construct.

Table S1: Comparative analysis of three *Bcl11b*-targeted sgRNAs

Name	Sequence
sgLuc	ggcatttcgcagcctaccg
sgBcl11b#1	cgggttgccctgtttgcgg
sgBcl11b#2	gtggaggctaccatcctcg
sgBcl11b#3	ggtgagtagatcagggtcg

Table S2: Relative proportions and absolute cell numbers for scRNA Seq UMAP clusters and constructs

Celltype Cluster	im12328z1	im12328z1_sgBcl11b	iTom	sgBcl11b
**Absolute cell numbers**				
CD16 Mono_Cl:9	10	9	11	13
CD4 TCM_Cl:8	66	23	41	35
CD8 TEM_Cl:1	344	207	191	438
CD8 TEM_Cl:5	154	118	107	108
dnT_Cl:4	295	113	147	141
ILC_Cl:7	46	203	34	179
NK Proliferating_Cl:6	138	189	113	38
NK_Cl:0	225	669	142	424
NK_Cl:2	274	307	165	320
NK_Cl:3	220	380	213	146
**Relative cell frequencies**				
CD16 Mono_Cl:9	0,00564334	0,00405771	0,00945017	0,00705755
CD4 TCM_Cl:8	0,03724605	0,0103697	0,03522337	0,01900109
CD8 TEM_Cl:1	0,19413093	0,09332732	0,16408935	0,23778502
CD8 TEM_Cl:5	0,08690745	0,05320108	0,0919244	0,05863192
dnT_Cl:4	0,16647856	0,0509468	0,12628866	0,07654723
ILC_Cl:7	0,02595937	0,0915239	0,02920962	0,09717698
NK Proliferating_Cl:6	0,0778781	0,0852119	0,09707904	0,02062975
NK_Cl:0	0,12697517	0,30162308	0,12199313	0,23018458
NK_Cl:2	0,15462754	0,13841298	0,14175258	0,17372421
NK_Cl:3	0,1241535	0,17132552	0,18298969	0,07926167
